# Near-infrared *in vivo* imaging system for dynamic visualization of lung-colonizing bacteria in mouse pneumonia

**DOI:** 10.1128/spectrum.00828-24

**Published:** 2024-09-17

**Authors:** Daiki Yamaguchi, Go Kamoshida, Syun Kawakubo, Saki Azuma, Takamitsu Tsuji, Nobuo Kitada, Ryohei Saito-Moriya, Noriteru Yamada, Rentaro Tanaka, Ayane Okuda, Keisuke Ueyama, Shingo Isaka, Manaha Tomita, Ryuichi Nakano, Yuji Morita, Hisakazu Yano, Shojiro A. Maki, Kinnosuke Yahiro, Shinichi Kato

**Affiliations:** 1 Laboratory of Microbiology and Infection Control, Kyoto Pharmaceutical University, Kyoto, Japan; 2 Laboratory of Pharmacological and Experimental Therapeutics, Kyoto Pharmaceutical University, Kyoto, Japan; 3 Department of Infection Control Science, Meiji Pharmaceutical University, Tokyo, Japan; 4 Graduate School of Informatics and Engineering, The University of Electro-Communications, Chofu, Japan; 5 Department of Chemical and Biological Sciences, Faculty of Science, Japan Women’s University, Tokyo, Japan; 6 Department of Microbiology and Infectious Diseases, Nara Medical University, Nara, Japan; University of Miami, Miami, Florida, USA

**Keywords:** *In vivo *imaging, bacterial pneumonia, *Acinetobacter baumannii*, near-infrared bioluminescence, TokeOni, scientific complementary metal-oxide semiconductor (sCMOS) camera, antibacterial therapy

## Abstract

*In vivo* imaging of bacterial infection models enables noninvasive and temporal analysis of individuals, enhancing our understanding of infectious disease pathogenesis. Conventional *in vivo* imaging methods for bacterial infection models involve the insertion of the bacterial luciferase LuxCDABE into the bacterial genome, followed by imaging using an expensive ultrasensitive charge-coupled device (CCD) camera. However, issues such as limited light penetration into the body and lack of versatility have been encountered. We focused on near-infrared (NIR) light, which penetrates the body effectively, and attempted to establish an *in vivo* imaging method to evaluate the number of lung-colonizing bacteria during the course of bacterial pneumonia. This was achieved by employing a novel versatile system that combines plasmid-expressing firefly luciferase bacteria, NIR substrate, and an inexpensive, scientific complementary metal-oxide semiconductor (sCMOS) camera. The D-luciferin derivative “TokeOni,” capable of emitting NIR bioluminescence, was utilized in a mouse lung infection model of *Acinetobacter baumannii*, an opportunistic pathogen that causes pneumonia and is a concern due to drug resistance. TokeOni exhibited the highest sensitivity in detecting bacteria colonizing the mouse lungs compared with other detection systems such as LuxCDABE, enabling the monitoring of changes in bacterial numbers over time and the assessment of antimicrobial agent efficacy. Additionally, it was effective in detecting *A. baumannii* clinical isolates and *Klebsiella pneumoniae*. The results of this study are expected to be used in the analysis of animal models of infectious diseases for assessing the efficacy of therapeutic agents and understanding disease pathogenesis.

**IMPORTANCE:**

Conventional animal models of infectious diseases have traditionally relied upon average assessments involving numerous individuals, meaning they do not directly reflect changes in the pathology of an individual. Moreover, in recent years, ethical concerns have resulted in the demand to reduce the number of animals used in such models. Although *in vivo* imaging offers an effective approach for longitudinally evaluating the pathogenesis of infectious diseases in individual animals, a standardized method has not yet been established. To our knowledge, this study is the first to develop a highly versatile *in vivo* pulmonary bacterial quantification system utilizing near-infrared luminescence, plasmid-mediated expression of firefly luciferase in bacteria, and a scientific complementary metal-oxide semiconductor camera. Our research holds promise as a useful tool for assessing the efficacy of therapeutic drugs and pathogenesis of infectious diseases.

## INTRODUCTION

Evaluating pathogenicity in *in vivo* animal models of bacterial infections is crucial for gaining a comprehensive understanding of the landscape of infectious diseases. The progression of infectious diseases undergoes dynamic changes from the initial stages to either death or recovery. Observing the pathogenesis of infectious diseases within a host organism over time can reveal intricate mechanisms of pathogenesis and disease exacerbation. Host colonization and proliferation are important steps in the early stages of infectious disease pathogenesis and closely related to the progression of the disease (1, 2). Therefore, temporal monitoring of the number of organ-associated bacteria is helpful for understanding the pathogenesis of infectious diseases and evaluating the efficacy of therapeutic agents. *In vivo* animal models of infection are commonly analyzed by sacrificing animals at a specific time point after infection and performing invasive analyses, such as assessment of the number of bacteria colonizing tissues, pathological analysis of tissue sections, or analysis of the inflammatory properties of blood samples or organs. However, these methods predominantly rely on the average assessment of numerous individuals at specific time points and lack the ability to directly reflect changes over time in the same individual (3, 4). Furthermore, in recent years, ethical considerations have led to a global demand to reduce the number of animals used in model systems (5, 6). One way to address these issues is the use of *in vivo* imaging, which enables noninvasive and temporal assessment of changes in the number of colonizing bacteria within the same individual, thereby minimizing inter-individual variability and reducing the number of animals used (5, 7).

In contrast to radiation-based systems such as X-rays, those using fluorescence or luminescence are comparatively easier to implement, owing to fewer constraints on the location of the detection device (8). Fluorescent systems involve fluorescent proteins and fluorophores of various wavelengths and are used for a variety of applications in both *in vitro* and *in vivo* evaluations. In fluorescent systems, an excitation light is emitted externally, and the resulting fluorescence is captured. However, because organs are located deep within the animal body, the photon intensity per centimeter is reduced to 1/10. Consequently, in fluorescent systems, both the excitation light and fluorescence are absorbed and significantly attenuated within the body (9, 10). Furthermore, autofluorescence also has to be considered. For these reasons, fluorescent systems cannot be used for the quantitative assessment of pathologies deep inside the body (10, 11). Therefore, the use of fluorescent systems for *in vivo* imaging of animal models to assess deep tissue pathology is difficult, and not only in models of bacterial infection. Although previous studies using bacterial infection models have reported detection of bacteria on the body surface, there are no reports on detecting bacterial colonization in deep organs, such as lung tissue, using only the fluorescent system (12–14). Meanwhile, bioluminescence does not require the irradiation of excitation light, and the likelihood of background luminescence is very low, making it less susceptible to noise (10, 15, 16). Hence, bioluminescence is generally used for *in vivo* imaging (10, 11). In particular, it is widely used as a method for analyzing tumor expansion, shrinkage, and metastasis in oncology (16–19).

*In vivo* imaging of models of bacterial infection is less developed than that in fields such as oncology, and there are no established methods that are both versatile and sensitive. The use of luminescent strains in which the luciferin-luciferase gene (*luxCDABE*) from marine bacteria is inserted into the genome has become the general bacterial imaging method due to the bacterial nature and simplicity of continuous bioluminescence (14, 20–29). The LuxCDABE system emits light at approximately 490 nm without requiring additional substrates (30). However, this method requires the cumbersome insertion of LuxCDABE into the genome, which limits its versatility. Additionally, the continuous luminescence reaction constantly depletes substances essential for bacterial survival (3, 31). This means that the luminescence signal is only proportional to the number of bacteria during the logarithmic growth phase (3, 27, 32). This LuxCDABE system is, thus, unsuitable for quantifying bacterial colonization in *in vivo* animal infection models. Additionally, wavelengths in the visible light spectrum below 600 nm are readily absorbed and scattered by hemoglobin and oxyhemoglobin, resulting in limited penetration into deep tissues; this decreases sensitivity when imaging deep tissues with high blood flow, such as the lungs (17, 18, 33).

Using longer wavelengths enhances light transmission through deep tissue. The wavelengths of the near-infrared (NIR) region (650–900 nm), called the “biological window,” demonstrates reduced absorption effects by biological substances and enhanced tissue permeability (17, 18, 33). Therefore, our focus shifted to D-luciferin derivatives, namely, “TokeOni” and “seMpai,” which, upon reacting with Luc2 (firefly luciferase), emit light in the NIR region with a maximum wavelength of approximately 670 nm. TokeOni and seMpai substantially enhance the sensitivity of *in vivo* imaging for tumor detection compared with D-luciferin (34, 35). However, no reports exist of *in vivo* imaging using NIR bioluminescence to monitor the number of colonizing bacteria during infection.

For *in vivo* imaging (including in the field of infectious diseases), a device with an ultra-sensitive charge-coupled device (CCD) camera is the most commonly used. However, CCD cameras are costly, posing a financial obstacle to their implementation. In recent years, *in vivo* imaging devices with inexpensive scientific complementary metal-oxide-semiconductor (sCMOS) cameras have become available. The use of sCMOS cameras is not common in *in vivo* imaging in the life sciences, and there are only a few reports on bioluminescence imaging (36). In particular, there are no reports on the *in vivo* imaging of bacterial infection models. Nevertheless, the use of sCMOS cameras could allow the widespread implementation of *in vivo* imaging to detect bacterial infections if the lack of sensitivity of sCMOS cameras compared with that of CCD cameras can be surmounted.

Antimicrobial-resistant bacterial infections pose a global threat to public health; therefore, there is a need to comprehend pathogenicity and devise novel antimicrobial agents. *A. baumannii* is globally recognized as a critical issue owing to its status as a drug-resistant bacterium, leading to its classification by the World Health Organization (WHO) and Centers for Disease Control and Prevention (CDC) as the highest-priority bacteria requiring new antibiotic development (37, 38). *A. baumannii* induces severe pneumonia in immunocompromised hosts and is difficult to treat due to its high fatality rate (39–41). In this study, we established a versatile *in vivo* imaging method using plasmid transduction, NIR bioluminescence, and an sCMOS camera based on an *A. baumannii* mouse pneumonia model. This system was able to temporally monitor the number of colonizing bacteria in the lungs. Our established *in vivo* imaging system is more sensitive and versatile than conventional methods, and we suggest that it could be applied to non-clinical studies such as those on antimicrobials.

## RESULTS AND DISCUSSION

### Highly sensitive visualization of lung-colonizing bacteria via *in vivo* NIR bioluminescence imaging

**Fig 1:**
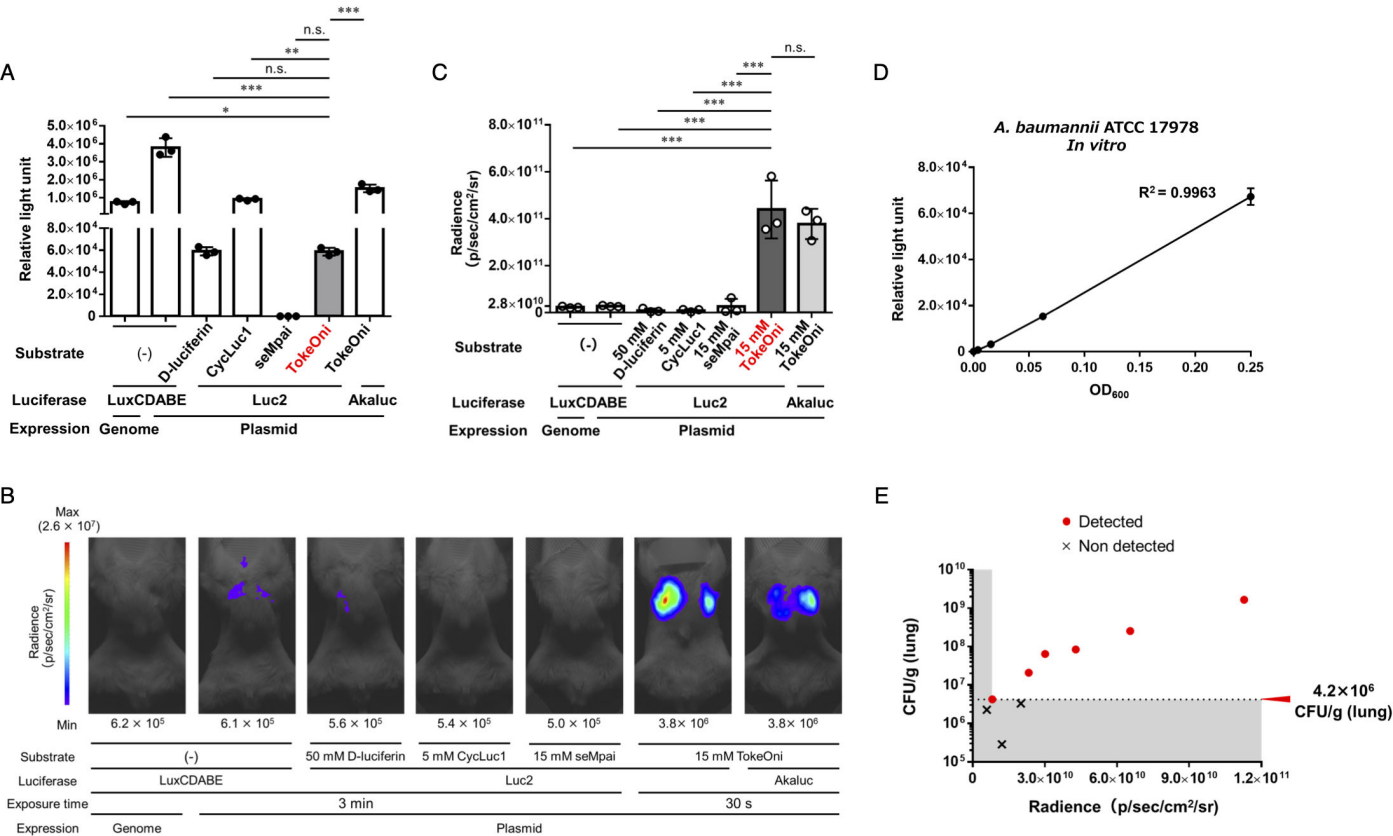
Optimization of a NIR emission imaging system for bacterial pneumonia. (**A**) A bacterial solution (ATCC 17978) with OD_600_ = 0.5 was prepared and mixed with an equal volume of saline for the LuxCDABE-expressing strain or an equal volume of 10 µM substrate for the Luc2- and Akaluc-expressing strains, and the luminescence signal was measured. Experiments were performed in triplicate. Mean values are shown and error bars indicate standard deviations (SDs). Immunodeficient mice were intratracheally administered with 5 × 10^7^ CFU/mouse luminescent-enzyme-expressing ATCC 17978 strains. *In vivo* imaging was conducted 24 h post-infection. Experiments were performed with three mice per group, and representative images are shown (**B**). The mean value of the luminescence signal obtained from all three images is shown (**C**). Means are indicated and error bars indicate SDs. (**D**) The linearity between the number of bacteria and luminescence signal was evaluated by mixing a 4-fold dilution series (OD_600_ = 0.5 to 4^−3^) of the bacterial solution (strain ATCC 17978-Luc) for measuring the luminescence signal. Experiments were independently performed in triplicate. Means are shown and error bars indicate SDs. (**E**) Immunodeficient mice were intratracheally administered with 1 × 10^7^ CFU/mouse of the ATCC 17978-Luc strain, and the bacterial load in the lungs was assessed. At 24 h post-infection, *in vivo* imaging quantification of lung-colonization bacteria was conducted using TokeOni. Experiments were performed on nine mice. Red dots represent plots of individuals for which luminescence was detected, and crosses indicate plots of individuals for which luminescence was not detected. Mean values are shown and error bars indicate SDs. ***P < 0.001, **P < 0.01, *P < 0.05 (One-way ANOVA and Dunnett’s test; **A, C**), n.s., not significant.

LuxCDABE is commonly utilized as a bioluminescence system for the *in vivo* imaging of bacteria; however, it lacks versatility, as the *luxCDABE* operon must be inserted into the genome. Therefore, we attempted to improve the versatility of luciferase expression by using a plasmid. First, plasmids expressing various luminescent enzymes [LuxCDABE, Luc2 (firefly luciferase), and Akaluc (firefly-luciferase mutant)] were constructed (Table 1) and subsequently transformed into the *A. baumannii* ATCC 17978 strain (Table 2). For comparison with the conventional method, a strain with LuxCDABE inserted into the genome was generated and transformed with the pKamoT-empty vector (Table 2). We investigated substrates emitting light at various wavelengths by reacting the Luc2-expressing ATCC 17978 strain (ATCC 17978-Luc) and comparing the *in vitro* luminescence intensity. D-luciferin and its derivative CycLuc1 emit light at approximately 560 nm and 600 nm, respectively, when they react with Luc2 (35). Akaluc, a firefly luciferase mutant enzyme, emits NIR luminescence at approximately 650 nm when exposed to TokeOni while demonstrating minimal reactivity with D-luciferin (42). Comparison of the *in vitro* luminescence intensities among various bioluminescence systems revealed that the luminescence signal of the LuxCDABE-plasmid-expressing strain exceeded 3 × 10^6^ relative light units (RLU). The luminescence signals of the ATCC 17978-Luc strain with CycLuc1 and LuxCDABE-genome-expressing strain were 9.0 × 10^5^ and 7.3 × 10^5^ RLU, respectively. By contrast, when seMpai was added to the ATCC 17978-Luc strain, the luminescence signal was 1.0 × 10^2^ RLU, showing nearly no luminescence. The luminescence signal of the ATCC 17978-Luc strain with TokeOni was 6.0 × 10^4^ RLU, which was over 1/10 lower than that of the LuxCDABE-expressing strain and CycLuc1-Luc2. The luminescence signal when TokeOni was added to the Akaluc-expressing strain was 1.5 × 10^6^ RLU, which was 25 times higher than the reaction between TokeOni and the ATCC 17978-Luc strain (Fig. 1A).

**TABLE 1:** List of plasmids

Plasmids	Ori	Antimicrobial for selection
pKamoT-empty	*E. coli* (ColE1), *Acinetobacter* species (pWH1266 ori)	Kanamycin, Tetracycline
pKamoT-luxCDABE		
pKamoT-luc2		
pHRPr-luc2	Broad host range ori for gram-negative bacteria (RSF1010)	Gentamycin

**TABLE 2:** List of luminescent strains

Strain		Plasmid	Abbreviations of luminescence strain
*Acinetobacter baumannii*	ATCC 17978	pKamoT-luc2	ATCC 17978-Luc
		pKamoT-luxCDABE	−
		pHRPr-luc2	ATCC 17978-GNLuc
	ATCC 17978-LuxCDABE (Genome expression)	pKamoT-empty	−
	ATCC 19606	pKamoT-luc2	−
	NR1127 (Clinical isolate)		NR1127-Luc
	NR4001 (Clinical isolate)		NR4001-Luc
*Klebsiella pneumoniae*	ATCC 13883	pHRPr-luc2	Kp-GNLuc

To identify the optimal bioluminescence system for *in vivo* bacterial imaging, immunodeficient mice treated with cyclophosphamide were inoculated with various bioluminescent strains in the lungs, and luminescence was detected 24 h post-infection. A weak luminescence of 2.8 × 10^10^ p/sec/cm^2^/sr was observed in the lungs of LuxCDABE-plasmid-expressing strain-inoculated mice, whereas luminescence was barely detected when mice were given in the presence of the LuxCDABE-genome expression strain and D-luciferin, CycLuc1, or seMpai in the presence of the ATCC 17978-Luc strain (Fig. 1B and C; Fig. S1A). Thereafter, we administered TokeOni as a substrate to the ATCC 17978-Luc-strain-inoculated mice and detected strong luminescence of colonized bacteria in the lungs (4.4 × 10^11^ p/sec/cm^2^/sr), achieving a significant 16-fold increase in sensitivity compared with the LuxCDABE-expressing strain (Fig. 1C). As previously mentioned, wavelengths below 600 nm are readily absorbed and scattered by substances in the body, resulting in poor tissue permeability (17, 18, 33, 35). These results, therefore, suggest that the enhanced sensitivity observed in the *in vivo* imaging of bacterial pneumonia can be attributed to the effective suppression of absorption and scattering by using NIR emission. The luminescence signal in mice inoculated with the Akaluc-expressing strain and TokeOni was approximately 3.8 × 10^11^ p/sec/cm^2^/sr (Fig. 1B and C;Fig. S1A). This was comparable with the luminescence intensity observed when TokeOni was administered to a group inoculated with the ATCC 17978-Luc strain. The TokeOni–Akaluc pair demonstrated a markedly increased sensitivity (both *in vitro* and *in vivo*) within an experimental system using mammalian cells and is currently one of the most sensitive experimental systems for *in vivo* imaging (42). The *in vitro* luminescence intensity of the Akaluc-expressing strain in bacteria was approximately 25-fold higher than that of Luc2. Notably, the *in vivo* luminescence intensities were comparable, indicating a discrepancy between *in vivo* and *in vitro* luminescence levels. This inconsistency may stem from the expression levels of Akaluc within the total bacteria in the host body, which necessitates further studies for a deeper understanding. These results suggest that the bioluminescence reaction of the TokeOni–Luc2 pair (TokeOni–Luc2 bioluminescence) can be used for *in vivo* imaging of bacterial pneumonia. The TokeOni-Luc2 system also achieved increased sensitivity compared with the LuxCDABE system, the conventional method. In the ATCC 19,606 *A*. *baumannii* strain, the luminescence signal was also detected in the lungs 24 h after bacterial infection using the TokeOni–Luc2 bioluminescence system (Fig. S1B).

The quantification and detection limits of TokeOni–Luc2 bioluminescence in the ATCC 17978-Luc strain were examined and a linear relationship (R^2^ = 0.9963) was observed between bacterial density and luminescence signal *in vitro* (Fig. 1D). To calculate the detection limit of the number of bacteria in the lungs using TokeOni–Luc2, immunodeficient mice were inoculated with low numbers of bacteria (1 × 10^7^ CFU/mouse). Twenty-four hours post-infection, we conducted *in vivo* imaging using the TokeOni–Luc2 system and counted the number of lung-colonizing bacteria. The results showed that the luminescence signal could be detected when the number of bacteria colonizing the lungs was 4.2 × 10^6^ CFU/g (lungs) or higher (Fig. 1E; Fig. S1C).

### Longitudinal monitoring of the dynamic changes of the number of bacteria in the lung and its application for evaluating the efficacy of antibacterial agents

**Fig 2:**
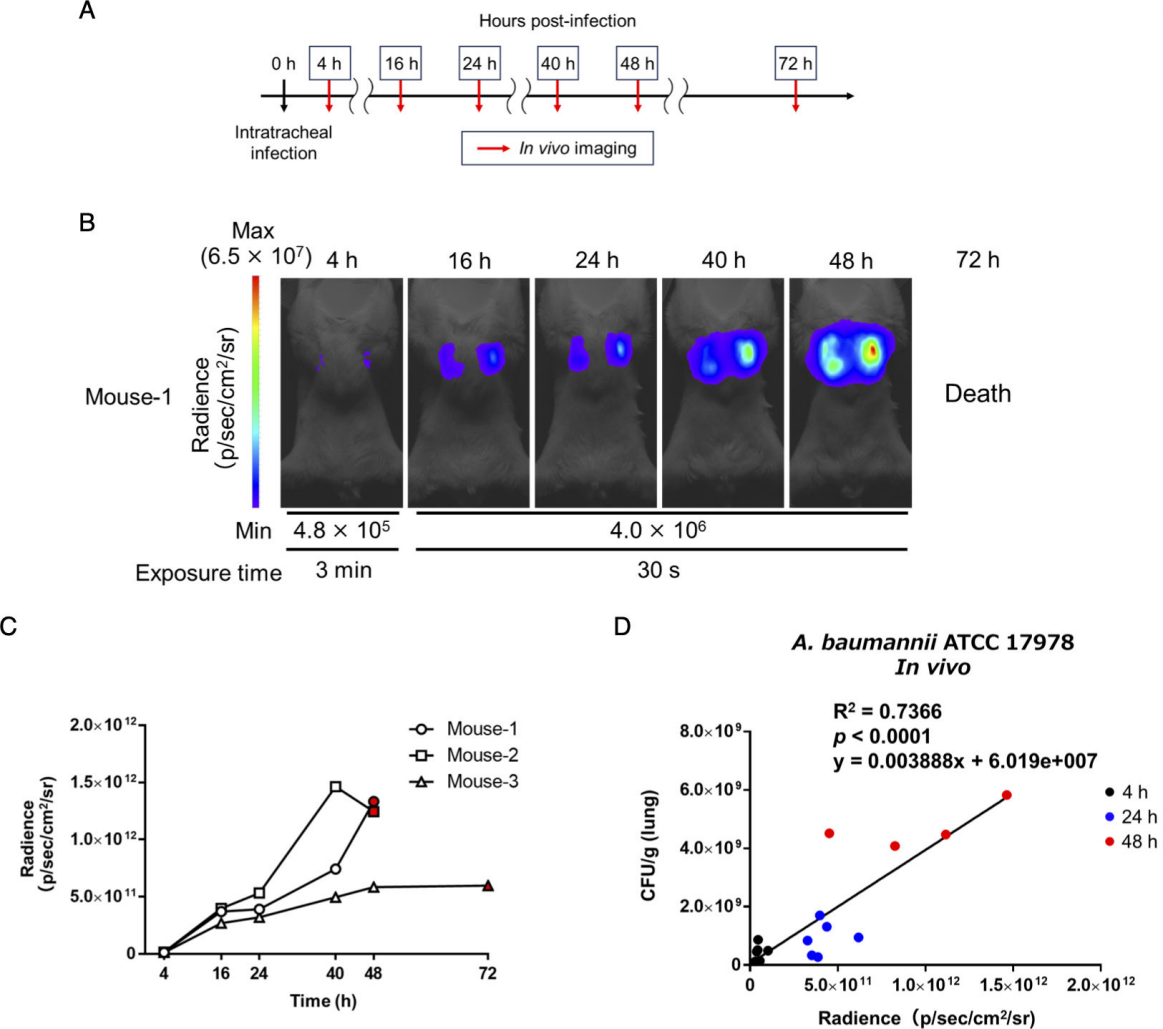
Temporal *in vivo* imaging of the number of lung-colonizing bacteria using TokeOni. (**A**) Immunodeficient mice were intratracheally administered ATCC 17978-Luc strain at a density of 5 × 10^7^ CFU/mouse, intraperitoneally administered TokeOni at the indicated time points post-infection, and *in vivo* imaging was performed. Experiments were performed on three mice per group. Representative images are shown in (**B**). Each signal extracted from all images was plotted for each individual imaging time point (**C**). The red symbols represent the results at the last time point that imaging was possible. (**D**) Immunodeficient mice were intratracheally administered 5 × 10^7^ CFU/mouse of the ATCC 17978-Luc strain, followed by intraperitoneal administration of TokeOni at 4, 24, and 48 h post-infection; *in vivo* imaging was then conducted. Six animals were used for each time point. At 48 h post-infection, two mice had died; hence, the experiment was performed with four surviving mice. The number of bacteria colonizing the lungs was measured after imaging at each time point, and the results were plotted alongside the corresponding image signals. The black, blue, and red symbols represent the results at 4, 24, and 48 h, respectively. The correlation between the luminescence signal in the lungs and the number of bacteria colonizing the lungs was evaluated. Correlation analysis was also performed using Pearson correlation coefficients, and the R^2^ and *P* values are shown on the graph.

**Fig 3:**
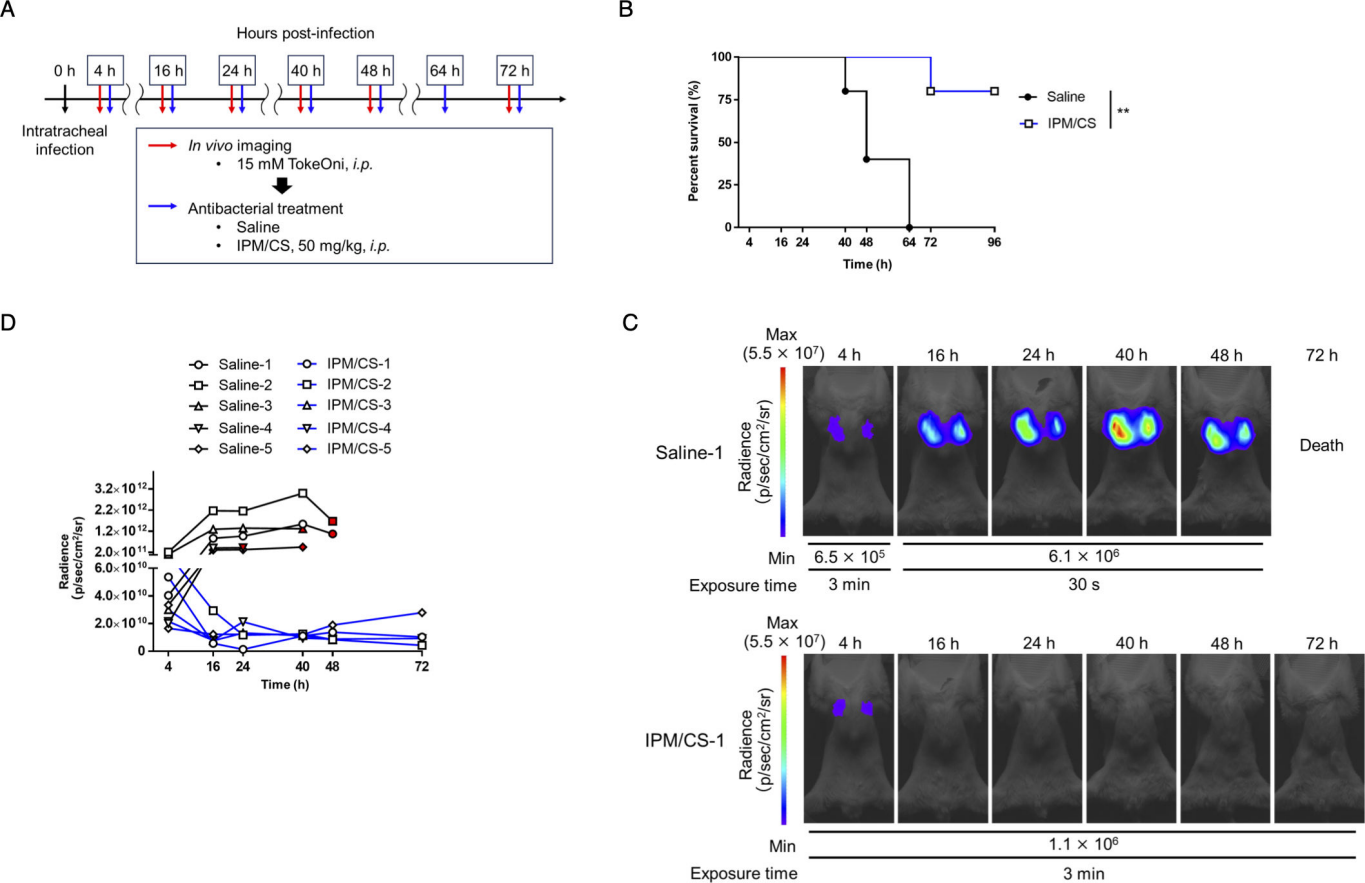
*In vivo* imaging for efficacy evaluation of antibacterial drugs using TokeOni. (**A**) Immunodeficient mice were intratracheally administered the ATCC 17978-Luc strain at a density of 5 × 10^7^ CFU/mouse. Subsequently, they were intraperitoneally administered TokeOni at the indicated time points, followed by *in vivo* imaging. Saline or imipenem/cilastatin (IPM/CS) was intraperitoneally administered at the indicated time points post-infection. Experiments were performed on five mice per group. (**B**) Kaplan–Meier plots. Representative images obtained via imaging are presented in (**C**). The signal obtained from all images was plotted for each imaging time point (**D**). The red symbols represent the results at the final imaging time point. ** *P* < 0.01 (Log-rank test; **B**).

The advantage of *in vivo* imaging is its capacity to noninvasively assess pathological changes within the same individual over time. In particular, monitoring the numbers of colonizing bacteria is one of the most crucial parameters in assessing the pathogenesis of infectious diseases and the efficacy of treatments. Thus, the ATCC 17978-Luc strain was used to infect cyclophosphamide-treated immunodeficient mice and bacterial colonization in the lungs from initial infection to death was monitored using *in vivo* imaging with TokeOni (Fig. 2A through C; Fig. S2). *In vivo* imaging and quantification of lung-colonizing bacteria were conducted to assess the correlation between the number of colonizing bacteria and the luminescence signal at 4, 24, and 48 h post-infection; a significant correlation (R^2^ = 0.7366, *P* < 0.0001) was observed (Fig. 2D). The obtained regression line was expressed by the formula y = 0.003888 × −6.019e + 007 (Fig. 2D). These results suggested that NIR bioluminescence imaging using TokeOni–Luc2 enables noninvasive and temporal visualization of the changes in the number of *A. baumannii* colonizing bacteria during lung infection within the same individual. Therefore, the imaging system was then used to monitor the number of lung-colonizing bacteria during antimicrobial therapy. As the ATCC 17978-Luc strain exhibited susceptibility to imipenem (Table 3), *in vivo* imaging with TokeOni was conducted in immunodeficient mice treated with saline or imipenem/cilastatin (IPM/CS) following pulmonary infection with the ATCC 17978-Luc strain (Fig. 3A). A luminescence signal in the lungs was initially detected in the IPM/CS-treated group; however, it subsequently disappeared, indicating a reduction in the number of bacteria colonizing the lungs that was associated with a tendency toward significant prolongation of survival (Fig. 3B through D; Fig. S3A). Conversely, light emission persisted in the saline-treated group and was associated with a higher mortality in these mice. Previous studies have used the LuxCDABE system to monitor the number of bacteria during treatment in a mouse pneumonia model of *Streptococcus pneumoniae* (20, 43). Notably, this study is the first to demonstrate *in vivo* imaging using *A. baumannii* to evaluate the effectiveness of therapeutic agents.

**TABLE 3:** MIC of luminescent strains

Agent (MIC units)	MIC for:			
	*A. baumannii*			*K. pneumoniae*
	ATCC 17978	NR1127	NR4001	ATCC 13883
	pKamoT-luc2			pHRPr-luc2
Imipenem /cilastatin (µg/mL）	0.25	0.0625	256	2
Levofloxacin (µg/mL）	0.25	0.5	0.125	0.03125

The number of bacteria administered in infection models varies greatly depending on the bacterial species. Typically, 1 × 10^7^ to 5 × 10^8^ CFU/mouse are administered intranasally or intratracheally in mouse pneumonia models using *A. baumannii*. In such cases, the number of bacteria colonized in the lungs 24 h post-infection was approximately 10^9^ to 10^10^ CFU/g (lung) in the non-treatment groups and approximately 10^6^ CFU/g (lung) in the antimicrobial agent-treatment groups (44–47). The number of lung-colonizing bacteria that can be reliably detected and linearly evaluated by our TokeOni–Luc2 system is predicted to be between 4.2 × 10^6^ and 6 × 10^9^ (Fig. 1E and 2D). Therefore, this system is not as sensitive as the conventional method of assessing bacterial numbers by culturing the homogenate tissue and counting the colonies, but it meets the required detection range for evaluating bacterial colonization in *A. baumannii* infection. The regression line (y = 0.003888 × −6.019e + 007, Fig. 2D) was used to estimate the amount of lung-colonizing bacteria at 4 h and 24 h in the non-treatment and treatment groups. The number of lung-colonizing bacteria in the treatment group was reduced by approximately 1,000-fold from 4 to 24 h post-infection; the number of lung-colonizing bacteria in the non-treated group at 24 h post-infection was approximately 5.7 × 10^2^ times higher than that in the treated group (Fig. S3B). These findings indicate that NIR bioluminescence imaging can be applied as a useful tool for evaluating antimicrobial therapies.

### Application of *A. baumannii* clinical isolates for assessing pulmonary infection status and the therapeutic efficacy of antimicrobial agents

**Fig 4:**
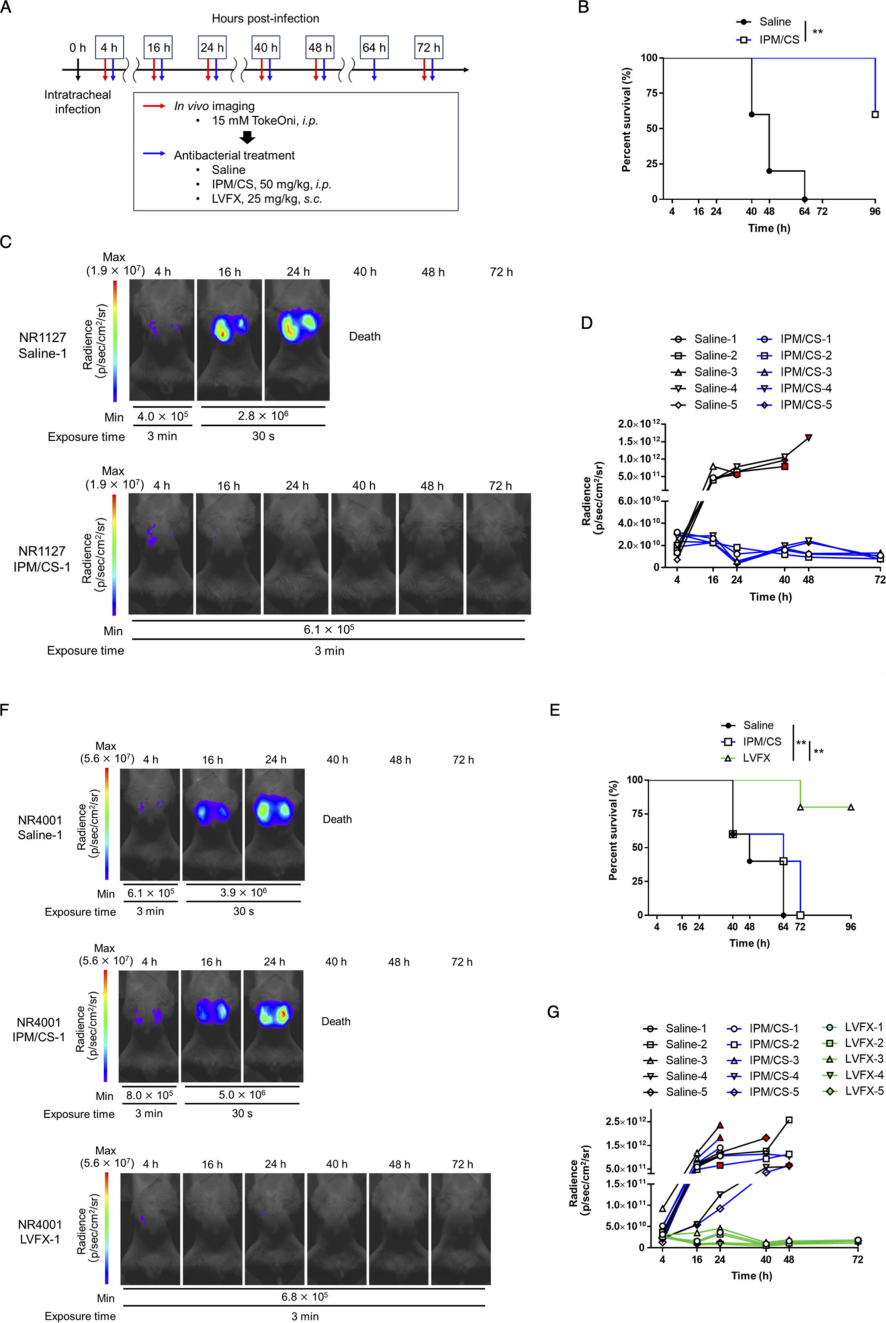
Evaluation of the number of lung-colonizing bacteria of *A. baumannii* clinical isolates and its application to the assessment of the therapeutic efficacy of antimicrobial agents. Clinical isolates of *A. baumannii* (NR1127-Luc, a carbapenem-sensitive strain, and NR4001-Luc, a carbapenem-resistant strain) were intratracheally administered to immunodeficient mice at 5 × 10^7^ and 1 × 10^8^ CFU/mouse, respectively. TokeOni was then administered intraperitoneally for *in vivo* imaging, and saline (*i.p.*), imipenem/cilastatin (IPM/CS) (*i.p.*), or levofloxacin (LVFX) (*s.c.*) was administered for treatment at the indicated time points post-infection. Experiments were performed using five mice per group (**A**). Kaplan–Meier plot of the NR1127-Luc strain (**B**) and NR4001-Luc strain (**E**). Representative images of the NR1127-Luc strain (**C**) and NR4001-Luc strain (**F**) acquired using *in vivo* imaging. The signal obtained from all images of the NR1127-Luc strain (**D**) and NR4001-Luc strain (**G**) was plotted for each time point. Red symbols represent the results at the final imaging time points.

The NIR bioluminescence imaging system developed in this study is plasmid- rather than chromosome-based, facilitating its adaptability to various strains. To assess the versatility of this system, we explored its feasibility for temporally evaluating the number of colonizing bacteria from clinical isolates of *A. baumannii*. Carbapenem-resistant *A. baumannii* is a global public health concern (37, 48, 49). Therefore, we established Luc2-expressing strains (NR1127-Luc and NR4001-Luc) using carbapenem-susceptible (NR1127) and carbapenem-resistant (NR4001) *A. baumannii* clinical isolates (Tables 2 and 3). In these strains, a linear relationship was observed between the luminescence signal *in vitro* and the density of the bacteria (NR1127-Luc, R^2^ = 0.9674; NR4001-Luc, R^2^ = 0.9928) (Fig. S4A and B). We detected the luminescence signals of the NR1127-Luc- or NR4001-Luc-strain-colonized lungs with *in vivo* imaging of immunodeficient mice using TokeOni (Fig. 4A). Thereafter, we investigated whether we could visualize the effect of antimicrobial treatment on lung infection over time using the NR1127-Luc or NR4001-Luc strains (Fig. 4B). As the NR4001-Luc strain exhibited resistance to IPM/CS, we explored alternative antibacterial agents distinct from kanamycin (aminoglycoside) and tetracycline (tetracycline), which serve as drug resistance markers for pKamoT-luc2. We found that the quinolone antimicrobial agent levofloxacin (LVFX) was effective against the NR4001-Luc strain (Table 3). *In vivo* imaging revealed that the luminescence signals in the lungs for both strains increased over time post-infection in the saline-treated groups, resulting in reduced mouse survival (Fig. 4B through G; Fig. S4C and D). With IPM/CS treatment, a lung luminescence signal was detected in the carbapenem-susceptible NR1127-Luc strain group at an early time point post-infection but subsequently disappeared; these mice exhibited significantly prolonged survival (Fig. 4B through D; Fig. S4C). Conversely, in mice inoculated with the carbapenem-resistant NR4001-Luc strain, the lung emission signal increased over time, ultimately resulting in reduced survival (Fig. 4E through G; Fig. S4D). In the LVFX-treated group, the luminescence signal of the NR4001-Luc strain did not increase from the early stages of infection and eventually disappeared, and it showed significantly prolonged survival compared with saline and IPM/CS-treated groups (Fig. 4E through G; Fig. S4D). These findings demonstrate the applicability of NIR bioluminescence imaging using TokeOni for clinical isolates of *A. baumannii*. This method enables noninvasive and temporal evaluation of the efficacy of therapeutic agents, reflecting the effectiveness of antimicrobial drugs *in vitro*.

The problem of antimicrobial resistance (AMR) affects the entire human population and *A. baumannii* serves as a representative example of AMR (37, 48, 49). Luc2 expression using a plasmid is easily achievable across various strains. However, the plasmids used in this study were resistant to kanamycin and tetracycline, limiting the number of strains suitable for transformation. Some *A. baumannii* strains are multidrug-resistant, having acquired resistance to various drugs (48, 50). In the future, we plan to employ plasmids containing a selection genes independent of antimicrobial resistance (51) to expand the range of transformed bacteria. Optimizing this system will enable the *in vivo* imaging of multidrug-resistant bacterial infections.

### Visualization of the clearance of lung-colonizing bacteria in immunocompetent hosts

**Fig 5:**
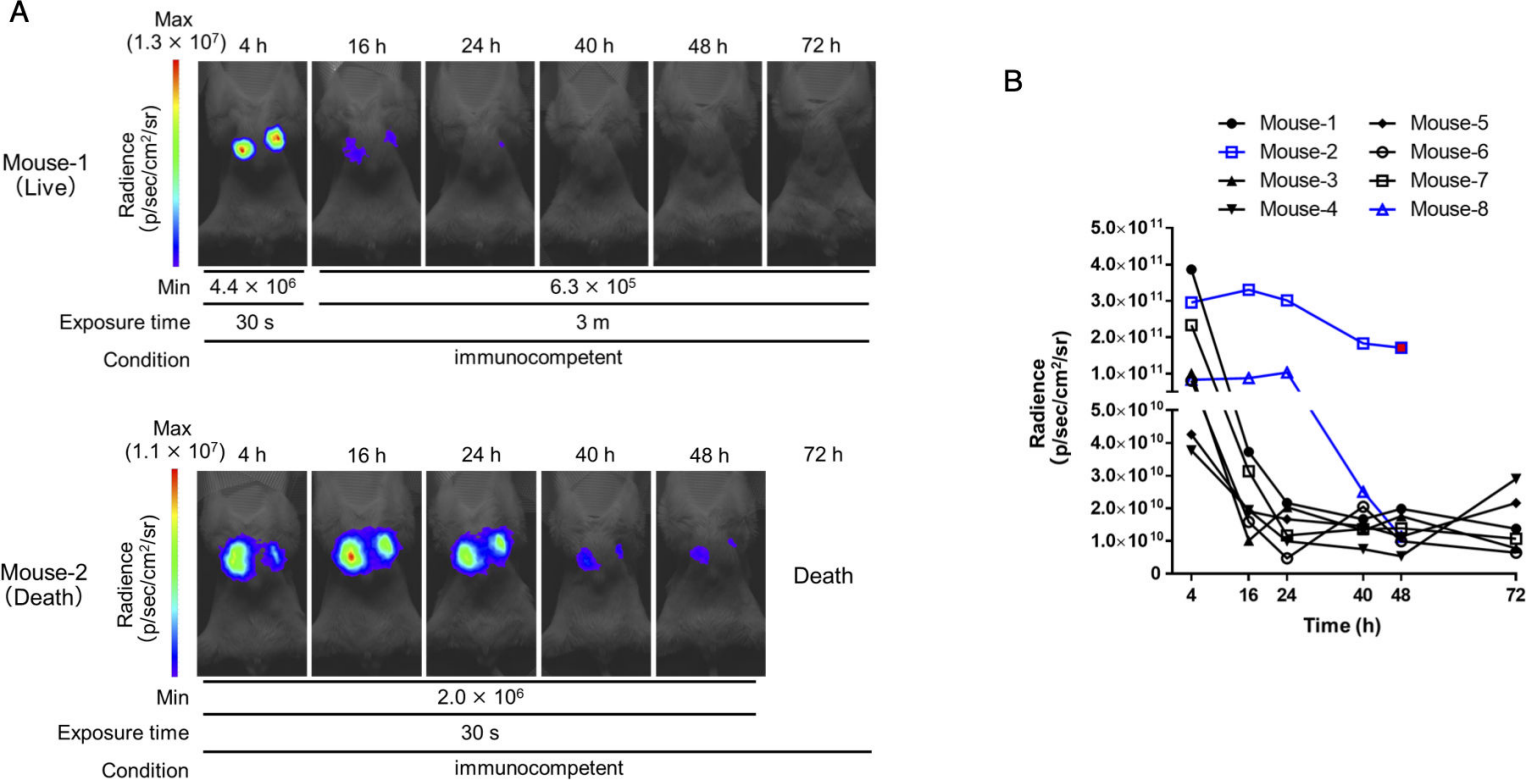
*In vivo* imaging of the clearance process of lung-colonizing bacteria in immunocompetent hosts. Immunocompetent mice were intratracheally administered ATCC 17978-Luc strain at a density of 5 × 10^8^ CFU/mouse. Subsequently, TokeOni was intraperitoneally administered at the indicated time points, followed by *in vivo* imaging. Experiments were performed on eight mice. Representative images of both surviving and deceased mice are shown in (**A**). The signal (*P*/sec/cm^2^/sr) obtained from all images was plotted for each time point (**B**). Black lines represent changes over time in surviving mice, whereas blue lines represent changes over time in deceased mice. Red dots indicate the results at the last imaging time point.

*A. baumannii* is an opportunistic pathogen that causes potentially fatal pneumonia and sepsis in immunocompromised patients in ICUs. However, *A. baumannii* is a typical opportunistic pathogen that is generally not pathogenic in immunocompetent hosts (39, 50). No reports exist of the *in vivo* imaging of *A. baumannii* infections in immunocompetent hosts over time. We investigated whether *in vivo* imaging could be used to visualize bacterial colonization and clearance in immunocompetent hosts. In these experiments, 75% (6/8) of the mice exhibited a decrease in luminescence signal over time from the early stage of infection, the luminescence from the bacteria finally disappeared, and the mice survived (Fig. 5A and B; Fig. S5). These results indicate that this system can be used to visualize the clearance process in immunocompetent hosts. Notably, 25% (2/8) of the *A. baumannii*-inoculated mice that succumbed to infection maintained a stronger luminescence signal up to 24 h post-infection compared with the surviving mice. Subsequently, the signal decreased, and the mice eventually died (Fig. 5A and B; Fig. S5). The luminescence signals in the surviving mice at 4 h post-infection were higher than those in the mice that succumbed to infection; however, these higher signals decreased and disappeared from 16 to 24 h post-infection (Fig. 5B). When treatment of immunodeficient mice with IPM/CS for lung infection was started 24 h post-infection (Fig. S3C), survival was significantly prolonged compared with the saline-treated group (Fig. S3D), but all mice died within 72 h. The luminescence signal in the lungs decreased after 48 h compared with 24 h post-infection and was below the limit of detection (Fig. S3E and F). These results suggest the importance for survival of treatment at an early stage of infection to reduce bacterial numbers in the *A. baumannii* lung infection model. In this study, we assessed pathogenesis using a model mimicking opportunistic infections based on differences in the immune conditions of the host. However, despite a reduction in the bacterial load, some mice died, indicating that mortality factors such as inflammation and sepsis, which cannot be solely attributed to a decrease in lung-colonizing bacteria, are pertinent. In future investigations, using animal models with underlying conditions such as tumors or diabetes, which are associated with a higher risk of infectious disease onset, or *in vivo* imaging systems for visualizing inflammatory conditions will more accurately reflect host pathophysiology and allow infection assessment in models resembling clinical scenarios (52–54).

### Assessment of the number of lung-colonization bacteria over time using *K. pneumoniae*

**Fig 6:**
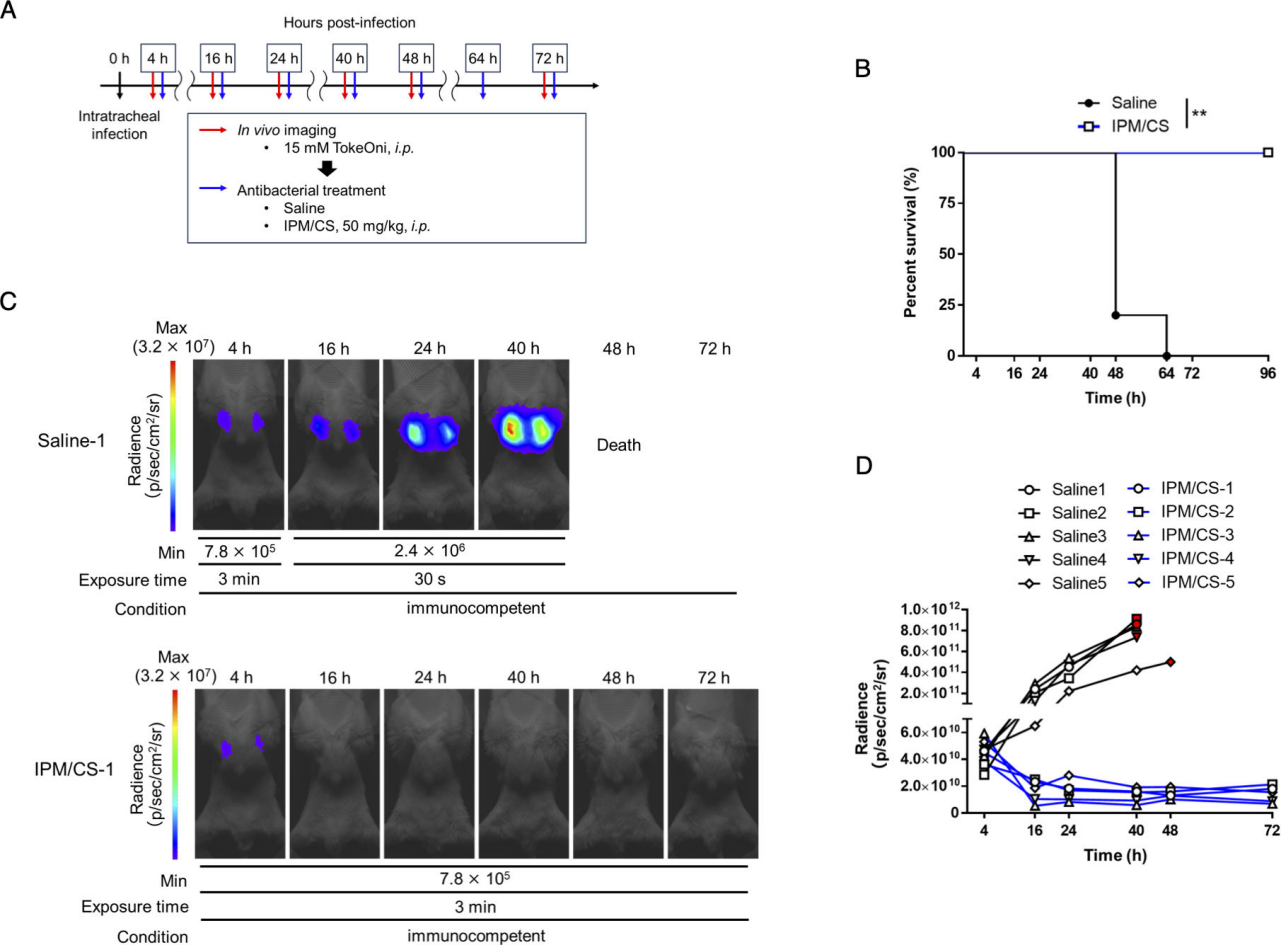
*In vivo* imaging of lung-colonizing bacteria in *K. pneumoniae* pneumonia. (**A**) Immunocompetent mice were intratracheally administered the Kp-GNLuc strain at a density of 5 × 10^8^ CFU/mouse. TokeOni was then administered intraperitoneally for *in vivo* imaging, and saline or imipenem/cilastatin (IPM/CS) was intraperitoneally administered for treatment at the indicated time points post-infection. Experiments were performed using five mice per group. (**B**) Kaplan–Meier plots. Representative images obtained via imaging are shown in (**C**). The signal obtained from all images was plotted for each time point (**D**). Red symbols represent the results at the final imaging time points. ** *P* < 0.01 (Log-rank test; **B**).

The use of plasmids is anticipated to facilitate the *in vivo* imaging of various bacteria, expanding beyond the species evaluated in existing reports. The species into which the plasmid can be transformed is defined by the origin of replication (ori). A plasmid carrying the RSF1010 ori can be transformed into a broad range of gram-negative bacteria (55). Therefore, we constructed a new plasmid (pHRPr-luc2) that expresses Luc2 and contains the RSF1010 ori. *K. pneumoniae* is a species of carbapenem-resistant *Enterobacterales* classified as the most critical bacterium by the WHO and CDC, similar to carbapenem-resistant *A. baumannii* (37, 38). *K. pneumoniae* ATCC 13883 (Kp-GNLuc strain) was generated to evaluate whether the TokeOni–Luc2 system can be used in gram-negative bacteria other than *A. baumannii* (Table 2). Linearity (R^2^ = 0.9625) was observed between the luminescence signal of the Kp-GNLuc strain and the bacterial density *in vitro* (Fig. S6A). We also showed temporal imaging of lung infections in immunocompetent mice (Fig. S6B and C). Given that the Kp-GNLuc strain was susceptible to IPM/CS (Table 3), we examined whether the effect of IPM/CS treatment could be evaluated over time, as performed for *A. baumannii* (Fig. 6A). The results showed that the saline-treated group exhibited an increase in the luminescence signal from the initiation of infection to death. In contrast, in the IPM/CS-treated group, luminescence was detected in the early stage of infection but then disappeared and significantly prolonged survival compared with the saline group (Fig. 6B through D; Fig. S6D). These results indicated that NIR bioluminescence imaging using TokeOni is applicable to *K. pneumoniae*.

Introducing pHRPr-luc2 into *A. baumannii* ATCC 17978 (ATCC 17978-GNLuc strain) and conducting lung infection experiments in immunodeficient mice revealed that *in vivo* imaging using TokeOni at 24 h post-infection led to detectable luminescence in the lungs. However, the intensity of luminescence was significantly (*P* = 0.0346) lower than that of the ATCC 17978-Luc strain (Fig.S7A and B). There was no significant (*P* = 0.7823) difference in the number of lung-colonizing bacteria between the ATCC 17978-Luc and ATCC 17978-GNLuc strains (Fig. S7C), and *in vitro* luminescence intensity was significantly (*P* = 0.0021) lower in the ATCC 17978-GNLuc strain than in the ATCC 17978-Luc strain (Fig. S7D). The pWH1266 ori of pKamoT-luc2 and the RSF1010 ori of pHRPr-luc2 were approximately 60 and 20 copies, respectively (56, 57). Therefore, the decrease in luminescence signal was considered to be dependent on the number of copies of the plasmid, but further studies are needed to confirm this. Therefore, pKamoT-luc2 is more useful than pHRPr-luc2 as a plasmid for *A. baumannii*. Although the RSF1010 ori can only be transformed into gram-negative bacteria, the use of an appropriate ori should enable *in vivo* imaging using TokeOni–Luc2 for both gram-negative and gram-positive bacteria. In addition, the results suggested that it is important to select a plasmid that contains an appropriate ori and promoter for each bacterial species to achieve high sensitivity for a specific bacterial species.

A limitation of our study is the exclusive visualization of bacteria within the lungs during bacterial pneumonia. In pneumonia caused by *S. pneumoniae*, meningitis can occur owing to bacterial dissemination from the lungs through the bloodstream (58). In the *A. baumannii* mouse model, bacteria are observed in the kidneys owing to bacterial dissemination from the lungs (59). It is anticipated that the quantity of bacteria colonizing distant organs is lower than that in the lungs, which serve as the primary infection site. Our experiments indicated that the number of bacteria colonizing the liver, kidney, and spleen was approximately 1/1,000^th^ of that colonizing the lungs after bacterial pneumonia in mice (data not shown). Thus, to visualize bacteria in seeded organs, it will be important to increase the sensitivity of our system. Although there are few reports regarding the detection limits for similar methods and they have not used *A. baumannii*, the detection limits for lung colonization predicted in this study are comparable with those in other bacteria previously reported using a CCD camera (22, 24). This suggests that sensitivity can be increased by using a CCD camera. In addition, the substrates and enzymes used in this study were optimized for mammalian cells and not for bacteria. Therefore, the search for NIR substrates and luciferases optimized for bacteria will further increase sensitivity.

To our knowledge, this is the first report on the use of an NIR substrate “TokeOni,” a plasmid-based luciferase expression system, and an sCMOS camera to enhance the monitoring of the number of lung-colonizing bacteria over time. Furthermore, our system achieved increased sensitivity and versatility compared with conventional LuxCDABE-based *in vivo* imaging systems. Although sCMOS cameras are less sensitive than CCD cameras, they are inexpensive and could contribute to removing the financial barrier to the widespread *in vivo* imaging of bacterial infection models. With further improvements in sensitivity, sCMOS cameras could be employed as an auxiliary tool for the comprehensive evaluation of the systemic pathology of bacterial infections.

## MATERIALS AND METHODS

### Reagents

D-luciferin potassium salt (Biomedical Sciences Corporation, Tokyo, Japan) was dissolved in saline at a concentration of 50 mM and stored at −80°C. CycLuc1 (MedChemExpress Co., Ltd., Monmouth Junction, NJ, USA) was dissolved in 10% dimethyl sulfoxide (diluted in PBS) at 5 mM and stored at −80°C. seMpai was synthesized as previously reported (34), dissolved in phosphate-buffered saline (PBS) at 15 mM, and stored at −80°C. TokeOni was synthesized as previously reported (35), dissolved in saline at 15 mM, and stored at −80°C. IPM/CS (Sawai, Osaka, Japan) and LVFX (Daiichi Sankyo, Tokyo, Japan) were prepared in saline (Otsuka Pharmaceutical, Tokyo, Japan) at concentrations of 10 mg/mL and 5 mg/mL, respectively, and stored at −80°C. Cyclophosphamide (Fujifilm Wako Pure Chemical Industries, Osaka, Japan) was dissolved in saline at 20 mg/mL and stored at −80°C.

### Bacterial strains and growth conditions

*A. baumannii* ATCC 17978, ATCC 19606, and *K. pneumoniae* ATCC 13883 were purchased from ATCC (Manassas, VA, USA). The *A. baumannii* clinical isolates NR1127 and NR4001 were obtained from patients in Japan. Cultures were grown on Luria-Bertani (LB) medium (Nacalai Tesque Co., Ltd., Kyoto, Japan) at 37°C (135 rpm) for 18 h.

### Minimum inhibitory concentration assay

The minimum inhibitory concentrations (MICs) of the luminescent strains were determined using the microdilution method with LB medium, following a previously reported protocol (60).

### Preparation of expression plasmids and construction of bioluminescence strains

*Escherichia coli* HST02 (Takara Bio Inc., Shiga, Japan) was used to clone the luciferase expression plasmid. PrimeSTAR GXL Premix Fast (Takara Bio Inc.) was used for gene amplification via PCR. All primer sequences are listed in Table S1. The pKamoT vector contains a tetracycline promoter (tetP) upstream of the multi-cloning site. Luciferase expression plasmids were constructed as pKamoT-luc2 (Luc2), pKamoT-luxCDABE (LuxCDABE), and pKamoT-Akaluc (Akaluc). *luxCDABE* was amplified from pGEN-luxCDABE (Mr. Mobley, Addgene plasmid #44918) using the primers lux_F and lux_R. *Luc2* was amplified from pGL4.50 (Promega Corporation, Madison, WI, USA) using the primers luc2_F and luc2_R1. *Akaluc* was amplified using the primers luc2_F and aka_R from pcDNA3 Venus-Akaluc (provided by the RIKEN BRC through the National BioResource Project of the MEXT, Japan; cat. RDB 15781). In addition, pKamoT-empty was linearized using the primers pKamoT_F and pKamoT_R, and each luminescent gene was ligated using In-Fusion HD Cloning (Takara Bio Inc.). For construction of pKamoT vectors, selection was performed in the presence of 50 µg/mL of kanamycin and 5 µg/mL of tetracycline.

pHRPr-luc2 expressing Luc2, designed for transformation into a broad range of gram-negative bacteria, was assembled by ligating gene fragments amplified with the primers tetP_F and luc2_R2 (tetP and luc2) from pKamoT-luc2 and fragments amplified with the primers pHRP_F and pHRP_R from pHRP308 (RIKEN BRC). pHRPr-luc2 construction was performed in the presence of 10 µg/mL of gentamicin.

The insertion of *luxCDABE* into the chromosome of *A. baumannii* was performed according to a previously reported method (61). The insertion position in the genome was selected to be a terminator near the replication origin of the genome. First, the genome of *A. baumannii* ATCC 17978 was used as a template, and primers KAMO5_00040_F, KAMO5_00050_R, KAMO5_00060_F, and KAMO5_00060_R were used to amplify both sides of the insertion site by PCR. In addition, *luxCDABE* was amplified from pKamoT-luxCDABE, including the promoter, using primers tetP_F and luxE_R. In addition, the pSBKT5v2 vector (61) was linearized using primers pSBKT_F and pSBKT5v2_R. These four fragments were ligated using In-Fusion HD Cloning and transformed into *E. coli* HST02 strain to generate pSBKT6-ATCC 17978-tetP-luxCDABE. Next, pSBKT6-ATCC 17978-tetP-luxCDABE was transformed into *E. coli* S17-1 λpir and *luxCDABE* was inserted into the chromosome of *A. baumannii* ATCC 17978 using homologous recombination according to a previous report (61).

All transformations of *A. baumannii* were performed via electroporation. *A. baumannii* was cultured in LB medium for 18 h at 37°C (135 rpm). Thereafter, the culture was diluted 50-fold with fresh LB medium and incubated for 3 h until the logarithmic growth phase was reached. Subsequently, 3 mL of culture was washed twice with 1 mL 10% glycerol, and the resulting pellet was suspended in 60 µL 10% glycerol. One microgram of plasmid was added to the bacterial suspension and introduced via electroporation using GenePulser Xcell (Bio-Rad Laboratories, Inc., Hercules, CA, USA) in 1.8 kV, 25 µF, 200 Ω, and 1 mm cuvettes. The plasmids were incubated in SOC medium for 3 h at 37°C before plating on appropriate antimicrobial-containing medium.

The transformation of *K. pneumoniae* was conducted based on a previous report (62). *K. pneumoniae* ATCC 13883 was incubated for 18 h at 26°C (135 rpm), diluted 50-fold in LB medium containing 0.7 mM ethylenediaminetetraacetic acid (EDTA) to inhibit capsular formation, and subcultured for 2 h. Subsequent transformations were performed as previously described for *A. baumannii*.

### Comparison of luminescence intensity of luminescent strains *in vitro*

The luminescent strains were cultured for 18 h at 37°C (135 rpm) in LB medium supplemented with the appropriate antimicrobial agents. Subsequently, the strains were diluted 50-fold in fresh LB medium and incubated for 3 h until they reached the logarithmic growth phase. Thereafter, the bacterial solution was replaced with saline or PBS and adjusted to OD_600_ = 0.5. The Luc2- and Akaluc-expressing strains were suspended in an equal volume of 10 µM substrate (solvent corresponding to the substrate), and the luminescence signal was measured after a 10 second incubation. For LuxCDABE-expressing strains, the luminescence signal was directly measured by diluting the bacterial solution with an equal volume of saline. For the linearity assessment of the bacterial count and bioluminescence signal, a bacterial solution with OD_600_ = 0.5, prepared as previously described, underwent a 4-fold dilution series up to 4^−3^. The luminescence signal was measured using a GloMax® 20/20 Luminometer (Promega Corporation) with a one-second exposure time.

### Mice

All experiments were approved by the Kyoto Pharmaceutical University Animal Ethics Committee (approval no. A21-028) and conducted in accordance with the Guidelines for the Proper Conduct of Animal Experiments (Science Council of Japan). Six- to 8-week-old male BALB/c mice were purchased from Japan SLC Co. (Shizuoka, Japan) and housed under consistent temperature regulation and had *ad libitum* access to water and food. The lighting in the rearing room was cycled on and off every 12 h. Signs indicating substantial respiratory distress, such as respiratory urgency and reduced spontaneous breathing, along with symptoms such as prolonged recumbency and hunching or a notable weight loss of 20% or more were regarded as the humane endpoints.

### Lung infection

Immunodeficiency was induced via intraperitoneal (*i.p.*) administration of cyclophosphamide (150 mg/kg) at −7,–5, and −3 d before bacterial infection. Bacterial lung infection was conducted under anesthesia via *i.p.* administration of 0.75 mg/kg medetomidine (Nippon Zenyaku Kogyo Co. Ltd., Fukushima, Japan), 4.0 mg/kg midazolam (Maruishi Pharmaceutical Co. Ltd., Osaka, Japan), and 5.0 mg/kg butorphanol (Meiji Animal Health Co. Ltd., Tokyo, Japan). The bacterial load was introduced intratracheally into the lungs of mice using a 50 µL bacterial suspension. Noninvasive intratracheal administration was performed by orally inserting a 26 G cathelin needle (Top Co., Ltd., Tokyo, Japan) with a removed tip into the bronchus. Following infection, mice were revived via *i.p.* administration of 0.75 mg/kg atipamezole (Nippon Zenyaku Kogyo Co., Ltd.).

The bacterial strains were cultured for 20 h at 37°C with shaking at 135 rpm and subsequently washed twice with PBS. To infect immunodeficient mice, ATCC 17978-Luc strains were inoculated at a density of 1 × 10^7^ or 5 × 10^7^ CFU/mouse; NR1127-Luc, Ec-GNLuc, and Kp-GNLuc strains at a density of 5 × 10^7^ CFU/mouse; the NR4001-Luc strain at a density of 1 × 10^8^ CFU/mouse; and the ATCC 19606-Luc strain at a density of 5 × 10^8^ CFU/mouse. Immunocompetent mice were inoculated with ATCC 17978-Luc or Kp-GNLuc at a density of 5 × 10^8^ CFU/mouse.

### *In vivo* bioluminescence imaging

Prior to *in vivo* imaging, mice inoculated with Luc2-expressing strains were intraperitoneally injected with 100 µL of 50 mM D-luciferin, 5 mM CycLuc1, 15 mM seMpai, or 15 mM TokeOni. The Akaluc-expressing strains were intraperitoneally injected with 100 µL of 15 mM TokeOni. After 20 min of substrate administration, *in vivo* imaging was performed using the VISQUE InVivo Smart-LF (Vieworks Co., Ltd., Ansan, Korea) equipped with an sCMOS camera on a heated stage at 36°C under 2% isoflurane anesthesia. The device information and the imaging conditions are shown in Table S2. During imaging, both limbs of the mouse were taped so that the xiphoid process was visible on the skin and used as a landmark during image processing. Similarly, mice inoculated with LuxCDABE-expressing strains were cultured without substrate. *In vivo* temporal imaging was conducted 4, 16, 24, 40, 48, and 72 h post-infection. The exposure time of the acquired images was set appropriately, with a maximum of 3 min. The images were analyzed using CleVue software (Vieworks Co., Ltd.) under constant conditions (round filter: r = 5, area = 782.72 mm^2^) and the total signal (radiance [p/sec/cm^2^/sr]) was calculated.

### Correlation analysis of luminescence signal and lung-colonizing bacteria

For *in vivo* imaging 4, 24, and 48 h post-infection, 6 mice per group were intratracheally infected with 5 × 10^7^ CFU/mouse of ATCC 17978-Luc. Surviving mice were subsequently imaged *in vivo* using 100 µL of 15 mM TokeOni by intraperitoneal injection. After *in vivo* imaging, the bilateral lungs of the mice were extracted and homogenized in 250 µL of PBS to create a tissue suspension. Subsequently, a 10-fold dilution series of the tissue suspension was prepared and plated on LB agar medium containing 50 µg/mL kanamycin and 5 µg/mL tetracycline. LB agar was incubated for 20 h at 37°C, and the number of viable colonies was determined as the CFU. The corresponding luminescence signal is plotted on the x-axis, and the CFU/g of lung tissue is plotted on the y-axis. Pearson correlation analysis was used to assess the correlation between luminescence signal and the number of bacteria colonizing the lungs.

### Antibacterial therapy

Saline (100 µL, *i.p.*) served as the control, whereas IPM/CS (100 mg/kg of body weight/d, *i.p.*) and LVFX [50 mg/kg of body weight/d, subcutaneous injection (*s.c.*)] were used as antibacterial agents. Drugs were administered 4, 16, 24, 40, 48, 64, and 72 h post-infection or *in vivo* imaging. Experiments were also conducted in which IPM/CS was administered 24, 40, and 48 h post-infection in order to assess the timing of treatment initiation.

### Statistical analyses

Statistical analyses were performed using GraphPad Prism software (GraphPad Software, San Diego, CA, USA). All statistical data are expressed as the mean ± standard deviation (SD). Comparisons between the two groups were performed using an unpaired *t*-test. One-way ANOVA and Dunnett’s test were utilized for comparing multiple groups. A *P* value < 0.05 was considered a significant difference. Pearson correlation coefficients were used for correlation analysis. The log-rank test was conducted for the statistical analysis of the survival curves.

## SUPPLEMENTAL MATERIAL

Supplemental material spectrum.00828-24-s0001.docx Tables S1 and S2; Fig. S1 to S7.

ASM does not own the copyrights to Supplemental Material that may be linked to, or accessed through, an article. The authors have granted ASM a non-exclusive, world-wide license to publish the Supplemental Material files. Please contact the corresponding author directly for reuse.
