# Supplementary material for: Near-infrared in vivo imaging system for dynamic visualization of lung-colonizing bacteria in mouse pneumonia
Source: Microbiol Spectr. 2024 Sep 17;12(11):e00828-24. doi: 10.1128/spectrum.00828-24 (PMC11537041; doi:10.1128/spectrum.00828-24)
Supplement: Supplemental material — Tables S1 and S2; Fig. S1 to S7. [file spectrum.00828-24-s0001.docx]

| **Oligonucleotides** | **Sequences (5'→3')** |
| --- | --- |
| pKamoT_F | gtcgaccatctcagaagtgaaacgccgta |
| pKamoT_R | atgtatatctccttcttaaatctagaggatccacaggacgggt |
| luc2_F | gaaggagatatacatatggaagatgccaaaaacattaagaagggccca |
| luc2_R1 | tctgagatggtcgacttacacggcgatcttgccgcccttct |
| lux_F | gaaggagatatacatatgactaaaaaaatttcattcatta |
| lux_R | tctgagatggtcgactcaactatcaaacgcttcggttaag |
| aka_R | tctgagatggtcgacttacacggcgatcttgccgtccttcttggcctta |
| tetP_F | cgccgcggccgcagtgccaagcttgcatgcctgcaggagggtta |
| luc2_R2 | tctgagatggtcgacttacacggcgatcttgccgcccttct |
| pHRP_F | taagtcgaccatctcagattgtgacaatttaccgaacaactccgc |
| pHRP_R | actgcggccgcggcgaagcaggtgcgacagacgtcatac |
| KAMO5_00040_F | ttgaggggttttttgctgacaagctccgttaccataaaatta |
| KAMO5_00050_R | cctgcaggcatgcaattatctaaactttttgtggttttcattacga |
| KAMO5_00060_F | gcgtttgatagttgaagttactttgatctaaatcaa |
| KAMO5_00060_R | tacgaattcgagctcgcgaacgcatggatactccatgtg |
| tetP_F | ttgcatgcctgcaggagggttattgtctcatgagcgga |
| LuxE_R | tcaactatcaaacgcttcggttaag |
| pSBKT_F | gagctcgaattcgtaatcatgtca |
| pSBKT5v2_R | caaaaaacccctcaagacccgtttagaggccccaaggggttatgctag |

**Supplementary Table 1. List of primers**

| **Device information** |  |
| --- | --- |
| Sensor | 1.2” Backside illuminated sCMOS |
| Cooling | Thermoelectric Peltier Cooling |
| Resolution（HxV) | 1824 × 1824 |
| Pixel size | 6.5 μm × 6.5 μm |
| Detection Spectral Range | 220-940 nm |
| Quantum efficiency | Max. 94%; 550 nm， >85%; 450 nm-700 nm， >30%; 300 nm-950 nm |
| Digital Output | 16 bit |
| Readout noise | 1.6e- |
| SNR (Luminescence site max ROI  / Background max ROI) | 13.5 |
| **Imaging status** |  |
| Filter | Luminescence（220-940 nm） |
| Light | Off |
| Focus | 118 step |
| Iris（NA） | F1.8 |
| FOV | 15cm × 15cm |
| Camera Temperature | -25℃ |
| Stage Temperature | 36℃ |
| Type | Single-Frame（Frame count = 1） |
| Binning | 2 × 2 |
| HDR | HighGainMode |

**Supplementary Table 2. Device information and imaging conditions**

**
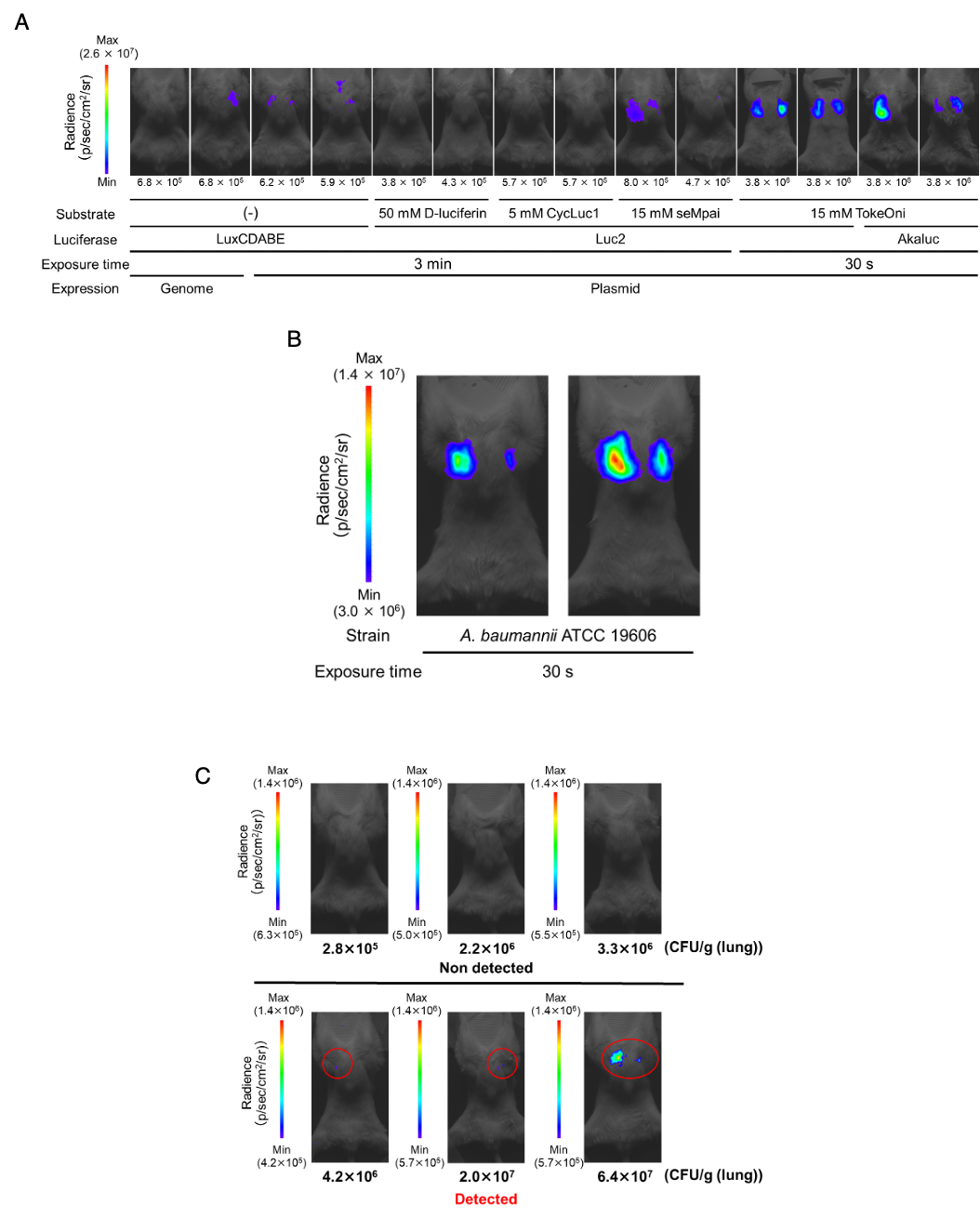
**

**Supplemental Figure 1. Optimization of a near-infrared (NIR) emission imaging system for bacterial pneumonia**

Immunodeficient mice were intratracheally administered 5 × 10^7^ CFU/mouse of various luminescent-enzyme-expressing strains of ATCC 17978 and *in vivo* imaging was conducted 24 h post-infection. Experiments were performed using three mice per group. Images of mice, except those shown in Figure 1B, are shown in (A). Immunodeficient mice were intratracheally administered 5 × 10^8^ CFU/mouse of the *A. baumannii* ATCC 19606 strain expressing Luc2. Twenty-four hours post-infection, TokeOni was intraperitoneally administered, and *in vivo* imaging was conducted. Experiments were performed on two mice, and the resulting images are shown in (B). To evaluate the detection limit of TokeOni-Luc, immunodeficient mice were intratracheally injected with the ATCC 17978-Luc strain at 1 × 10^7^ CFU/mouse. At 24 h post-infection, *in vivo* imaging was performed using TokeOni. After *in vivo* imaging, colony counts of lung-colonized bacteria were performed. Representative images near the limit of detection and the number of bacteria are shown (C).

**
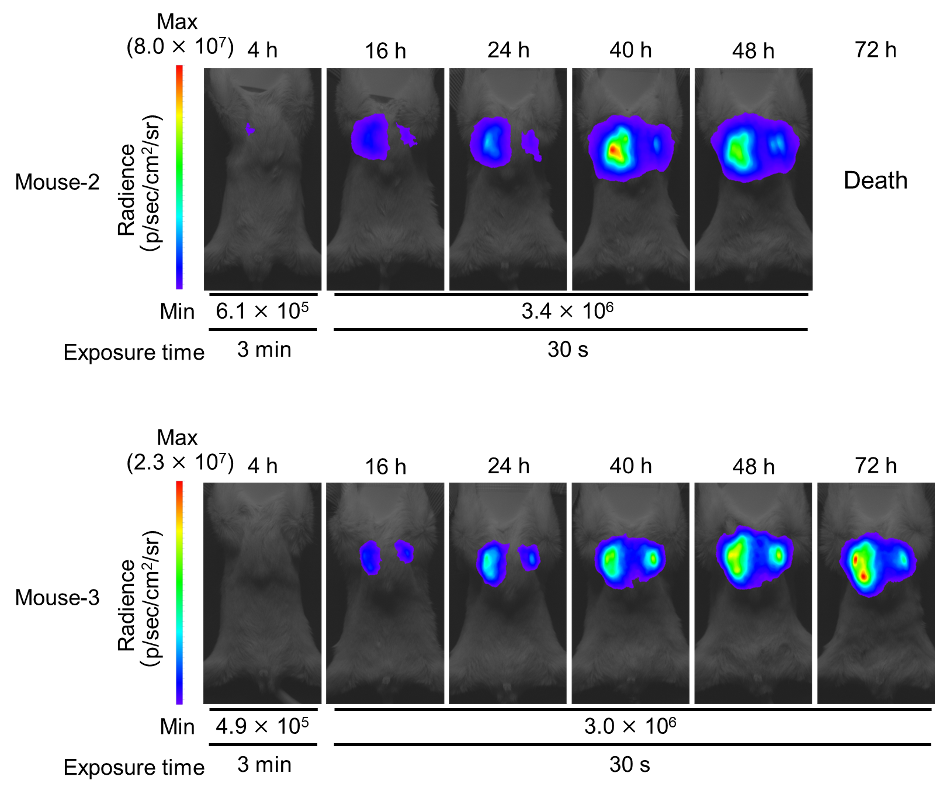
**

**Supplementary Figure 2. Temporal *in vivo* imaging of the number of lung-colonizing bacteria using TokeOni**

Immunodeficient mice were intratracheally administered the ATCC 17978-Luc strain at a density of 5 × 10^7^ CFU/mouse, intraperitoneally administered TokeOni at the indicated time points post-infection, and *in vivo* imaging was performed. Experiments were performed using three mice. Images of all mice, except those indicated in Figure 2B, are shown.

**
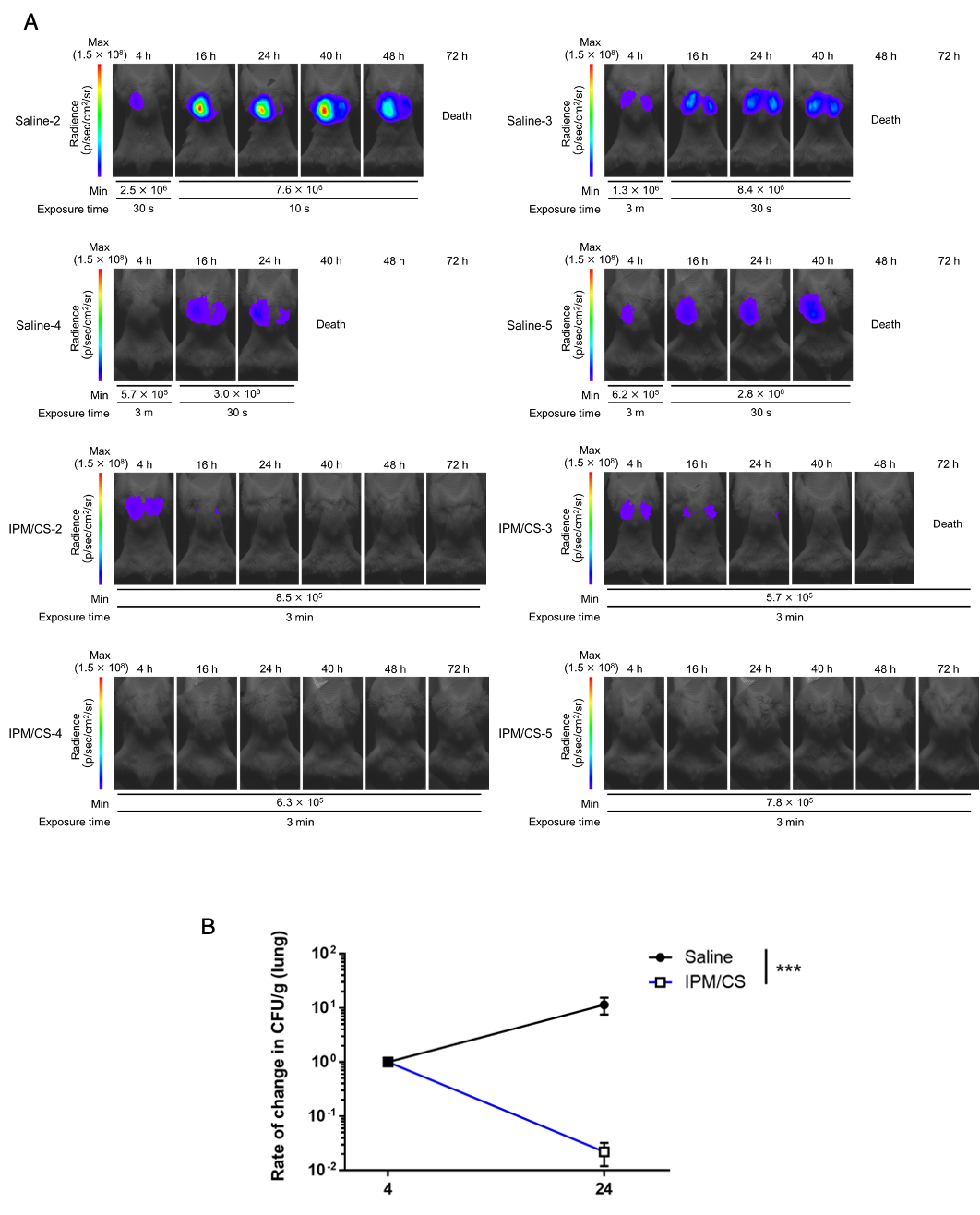
**

**
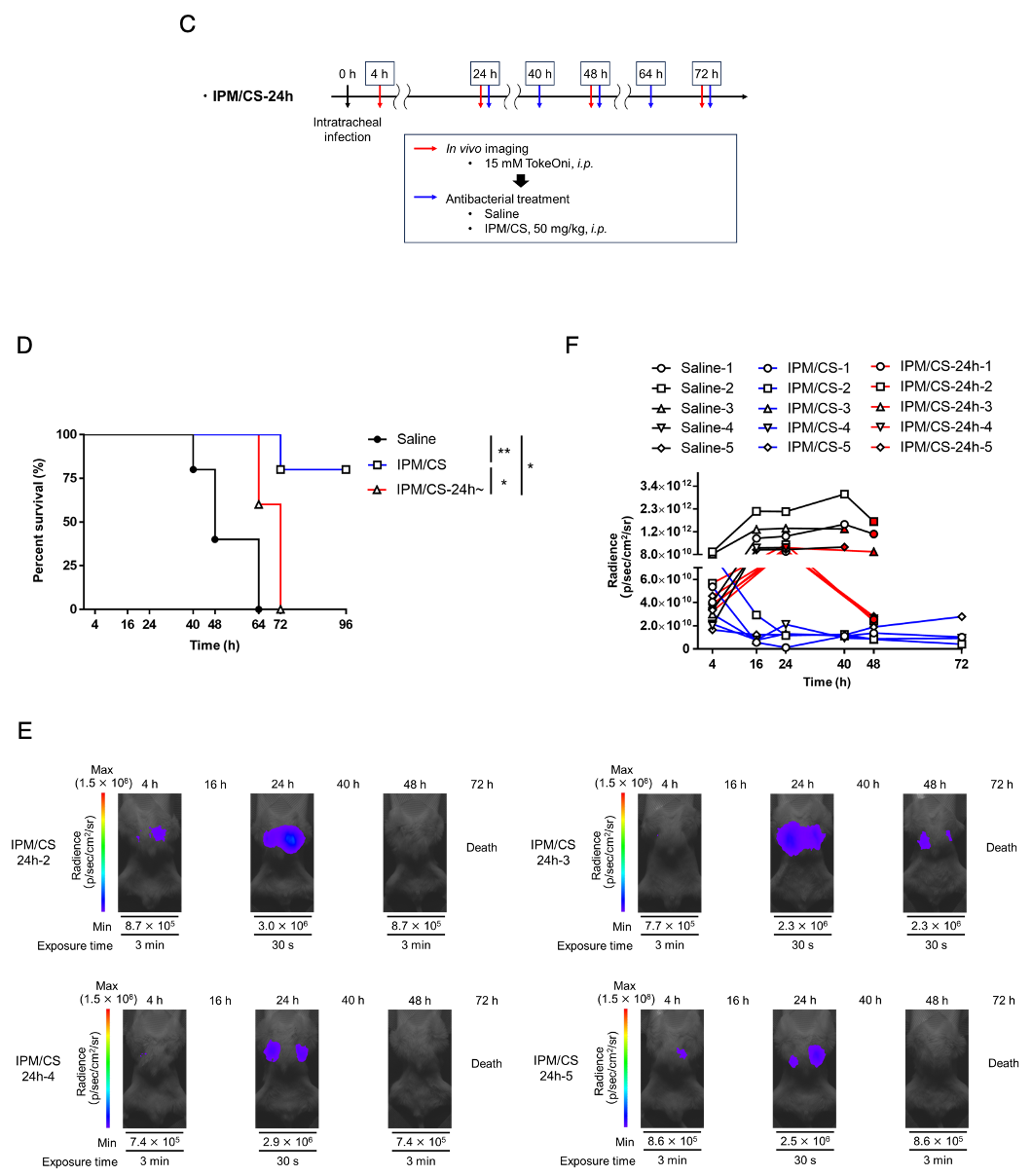
**

**Supplementary Figure 3. *In vivo* imaging for evaluation of antibacterial drug efficacy using TokeOni**

Immunodeficient mice were intratracheally administered ATCC 17978-Luc at a density of 5 × 10^7^ CFU/mouse. Subsequently, the mice were intraperitoneally administered TokeOni at the indicated time points post-infection. Saline or imipenem/cilastatin (IPM/CS) was intraperitoneally administered at the indicated time points post-infection. Experiments were performed using five mice per group. Images of the mice, except those indicated in Figure 3C, are shown (A). The number of lung-colonizing bacteria at 4 h and 24 h in both the non-treated and treated groups (n = 5) was estimated from the regression line, and the percentage change was calculated as expressed by the equation (y = 0.003888x - 6.019e + 007, Fig. 1E) (B). (C) Immunodeficient mice were intratracheally administered the ATCC 17978-Luc strain at a density of 5 × 10^7^ CFU/mouse. Subsequently, they were intraperitoneally administered TokeOni at the indicated time points, followed by *in vivo* imaging. Imipenem/cilastatin (IPM/CS) was intraperitoneally administered at the indicated time points post-infection. Experiments were performed on five mice. (D) Kaplan–Meier plots merged with Fig. 3B. Representative images obtained via imaging are presented in (E). (F) The signal obtained from all images was plotted for each imaging time point and merged with Fig. 3D. The red symbols represent the results at the final imaging time point. Mean values are shown and error bars indicate standard deviations (SDs). *** *p* < 0.001 (an unpaired *t*-test; B), ** *p* < 0.01, * *p* < 0.05. (Log-rank test; D).

**
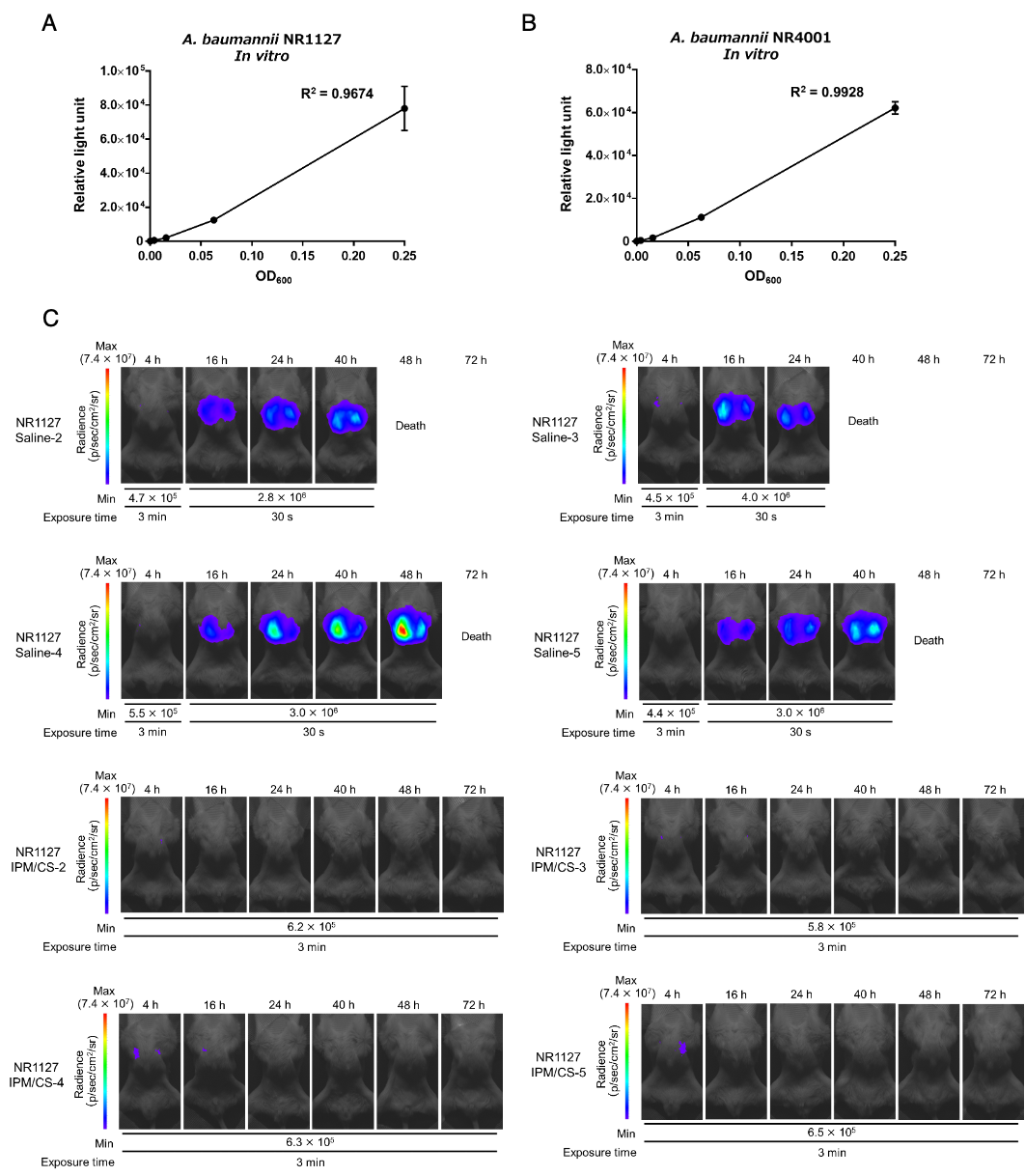
**

**
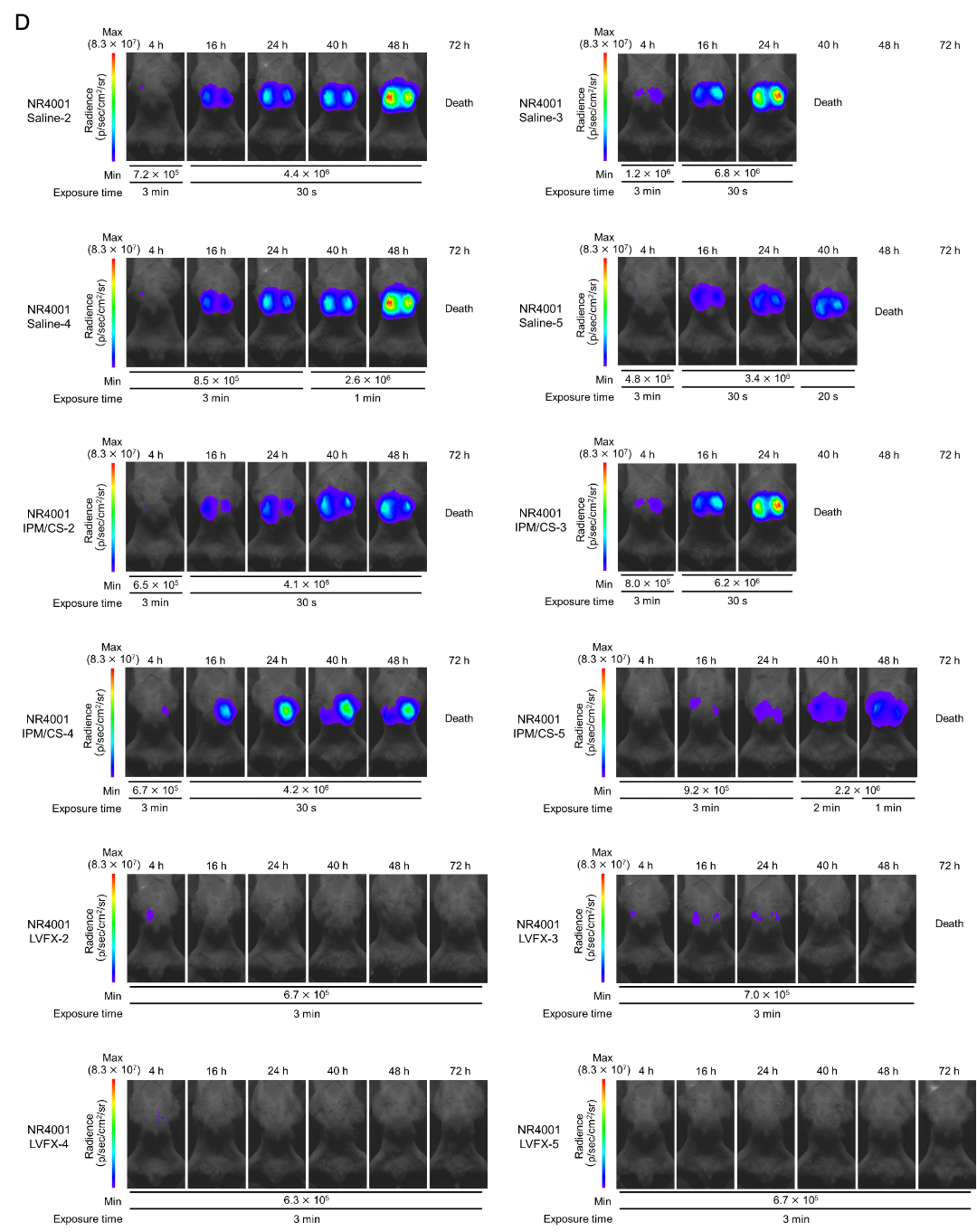
**

**Supplementary Figure 4. Evaluation of the number of lung-colonizing bacteria of *A. baumannii* clinical isolates and its application to the assessment of the therapeutic efficacy of antimicrobial agents**

The linearity between the number of bacteria and luminescence signal was evaluated by mixing a 4-fold dilution series (OD_600_ = 0.5 to 4^-3^) of the bacterial solution with TokeOni. Results are shown for the NR1127-Luc (A) and NR4001-Luc (B) strains. Experiments were independently performed in triplicate. Means are shown and error bars indicate standard deviations (SDs). The NR1127-Luc and NR4001-Luc strains were intratracheally administered to immunodeficient mice, followed by intraperitoneal administration of TokeOni at the indicated time points and *in vivo* imaging. Saline (*i.p.*), imipenem/cilastatin (IPM/CS) (*i.p.*), or levofloxacin (LVFX) (*s.c.*) was administered at the indicated time points post-infection. Experiments were performed using five mice per group. Results for the NR1127-Luc (C) and NR4001-Luc strains (D) are shown, except those indicated in Figure 4C, F.

**
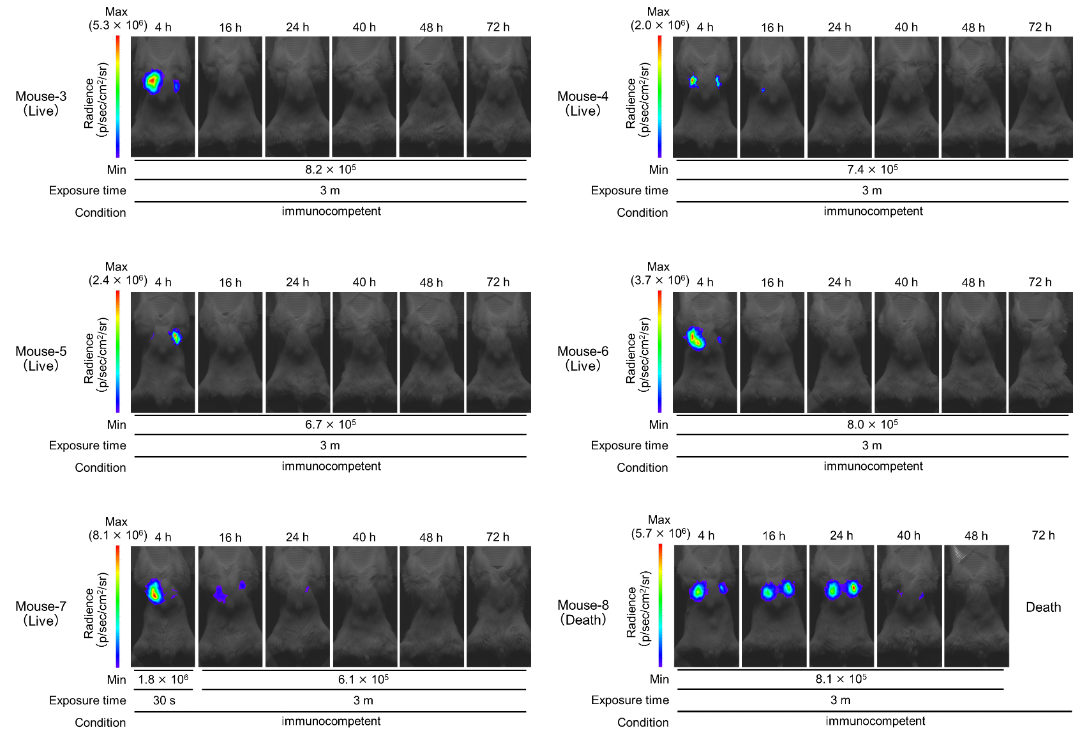
**

**Supplementary Figure 5. *In vivo* imaging of the clearance process of lung-colonizing bacteria in immunocompetent hosts**

Immunocompetent mice were intratracheally administered the ATCC 17978-Luc strain at a density of 5 × 10^8^ CFU/mouse. Subsequently, TokeOni was intraperitoneally administered at the indicated time points, followed by *in vivo* imaging. Experiments were performed using eight mice. Images of the mice, except those indicated in Figure 5A, are shown.

**
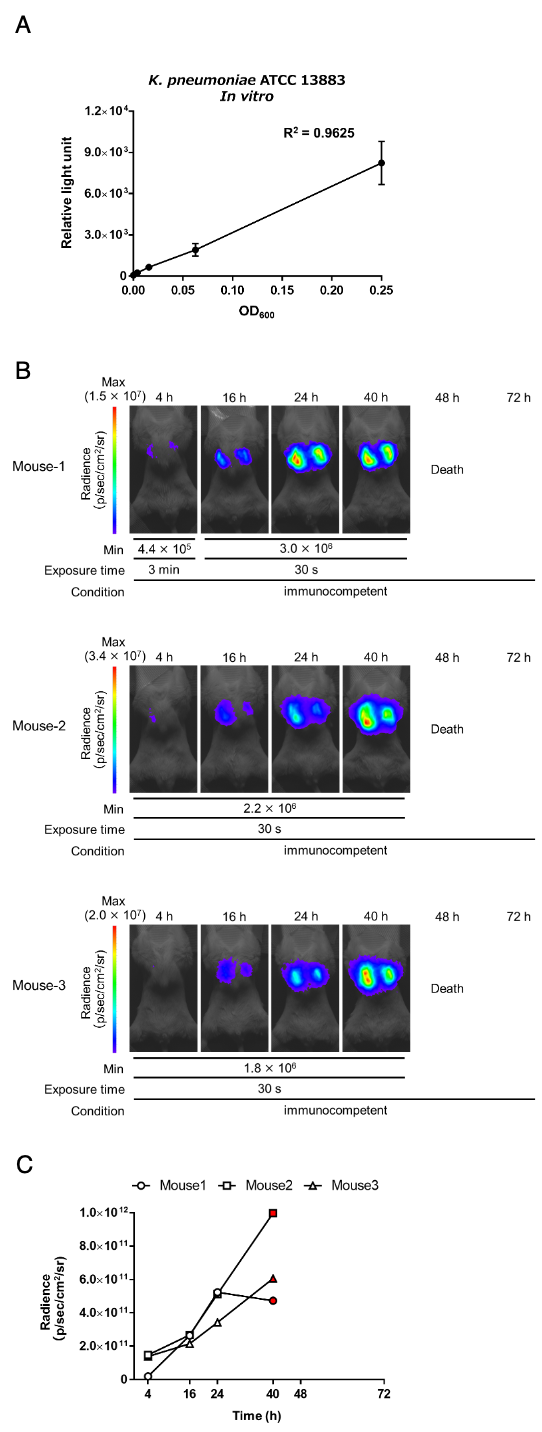
**

**
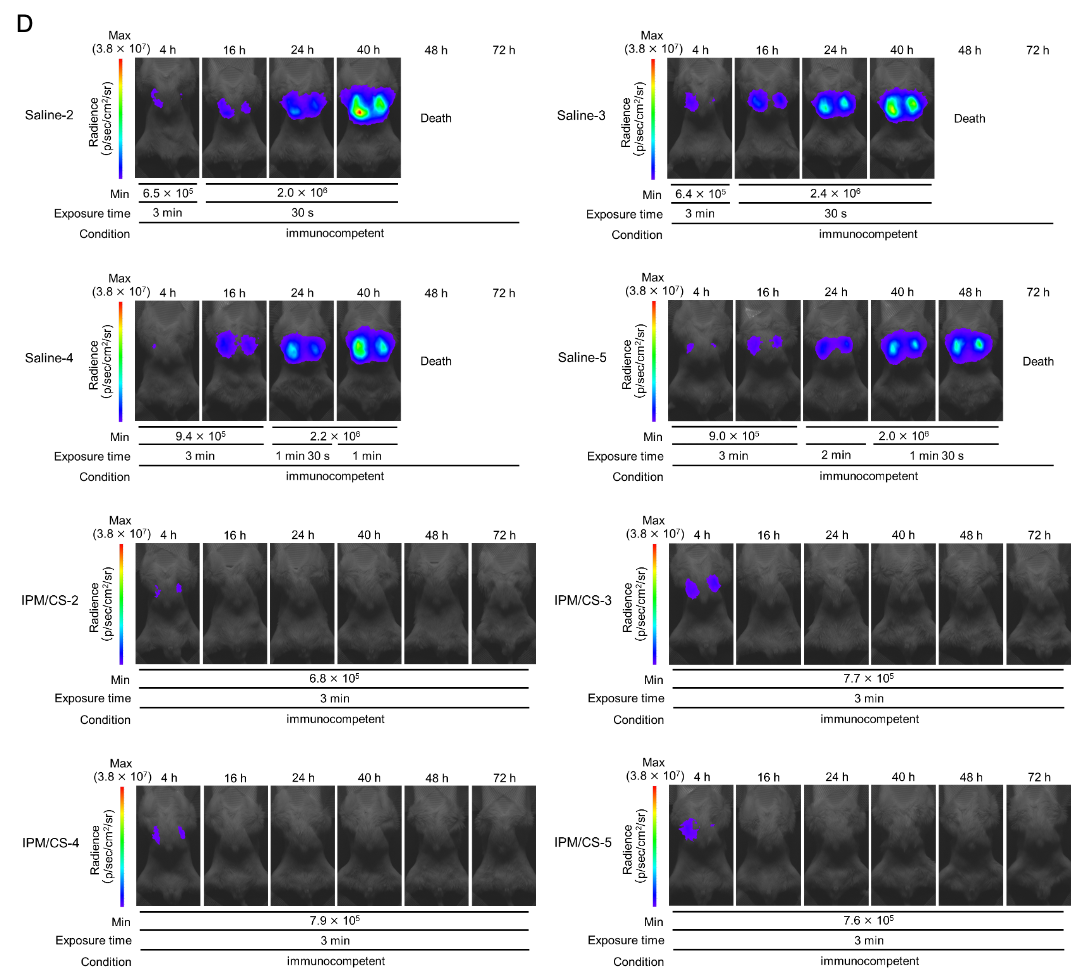
**

**Supplementary Figure 6. *In vivo* imaging of lung-colonizing bacteria in *Klebsiella pneumoniae* pneumonia**

(A) The linearity between the number of bacteria and luminescence signal was evaluated by mixing a 4-fold dilution series (OD_600_ = 0.5 to 4^-3^) of the bacterial solution (Kp-GNLuc strain) with TokeOni. Immunocompetent mice were intratracheally administered 5 × 10^8^ CFU/mouse of the Kp-GNLuc strain. Subsequently, the TokeOni was intraperitoneally administered at the indicated time points, followed by *in vivo* imaging. Experiments were performed using three mice. The signal obtained from all images was plotted for each imaging time point (C). Immunocompetent mice were intratracheally administered the Kp-GNLuc strain. Subsequently, TokeOni was intraperitoneally administered at the indicated time points, followed by *in vivo* imaging. Saline or imipenem/cilastatin (IPM/CS) was intraperitoneally administered at the indicated time points post-infection. Experiments were performed using five mice per group. Images of the mice, except those indicated in Figure 6C, are shown in (D).

**
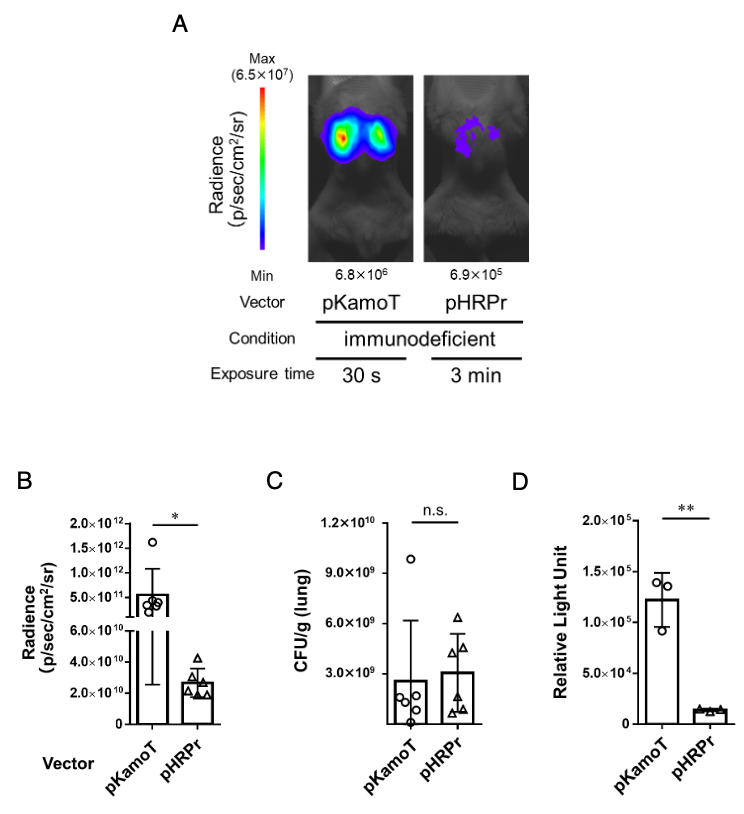
**

**Supplementary Figure 7. Comparison of pKamoT-luc2 and pHRPr-luc2 in *A. baumannii***

Immunodeficient mice were intratracheally administered the ATCC 17978-Luc or ATCC 17978-GNLuc strains. Twenty-four hours post-infection, TokeOni was intraperitoneally administered and *in vivo* imaging was conducted. Experiments were performed using six mice per group. Representative images obtained via imaging are presented in (A). The mean value per time point of the signal is shown. Error bars indicate standard deviations (SDs) (B). The measurement of the number of lung-colonizing bacteria was conducted after *in vivo* imaging, and the results are shown in (C). (D) A bacterial solution (ATCC 17978-Luc or ATCC 17978-GNLuc strains) with OD_600_ = 0.5 was mixed with an equal volume of 10 µM TokeOni and the luminescence signal was measured. Experiments were performed in triplicate. Mean values are shown, and error bars indicate standard deviations (SDs). ** *p* < 0.01, * *p* < 0.05 (an unpaired *t*-test; B, D), n.s., not significant.
